# Assessment of Reward-Related Brain Function After a Single Dose of Oxytocin in Autism: A Randomized Controlled Trial

**DOI:** 10.1016/j.bpsgos.2021.10.004

**Published:** 2021-10-23

**Authors:** Annalina V. Mayer, Katrin Preckel, Kristin Ihle, Fabian A. Piecha, Klaus Junghanns, Stefan Reiche, Lena Rademacher, Laura Müller-Pinzler, David S. Stolz, Inge Kamp-Becker, Sanna Stroth, Stefan Roepke, Charlotte Küpper, Veronika Engert, Tania Singer, Philipp Kanske, Frieder M. Paulus, Sören Krach

**Affiliations:** a Social Neuroscience Lab, Department of Psychiatry and Psychotherapy, University of Lübeck, Lübeck, Germany; b Translational Psychiatry Unit, Department of Psychiatry and Psychotherapy, University of Lübeck, Lübeck, Germany; c Max Planck Institute for Human Cognitive and Brain Sciences, Leipzig, Germany; d Department of Child and Adolescent Psychiatry, Psychosomatics and Psychotherapy, Philipps University of Marburg, Marburg, Germany; e Marburg Center for Mind, Brain and Behavior, Philipps University of Marburg, Marburg, Germany; f Department of Psychiatry and Psychotherapy, Charité – Universitätsmedizin Berlin, Campus Benjamin Franklin, Berlin, Germany; g Social Cognition Group, Berlin School of Mind and Brain, Department of Psychology, Humboldt-Universität zu Berlin, Berlin, Germany; h Social Neuroscience Lab, Max Planck Society, Berlin, Germany; i Institute of Psychosocial Medicine, Psychotherapy and Psychooncology, Jena University Hospital, Friedrich-Schiller University, Jena, Germany; j Clinical Psychology and Behavioral Neuroscience, Faculty of Psychology, Technische Universität Dresden, Dresden, Germany

**Keywords:** Amygdala, Autism spectrum disorders, Oxytocin, Reward, Social motivation, Ventral striatum

## Abstract

**Background:**

Autism spectrum disorder (ASD) is characterized by difficulties in social communication and interaction, which have been related to atypical neural processing of rewards, especially in the social domain. As intranasal oxytocin has been shown to modulate activation of the brain’s reward circuit, oxytocin might ameliorate the processing of social rewards in ASD and thus improve social difficulties.

**Methods:**

In this randomized, double-blind, placebo-controlled, crossover functional magnetic resonance imaging study, we examined effects of a 24-IU dose of intranasal oxytocin on reward-related brain function in 37 men with ASD without intellectual impairment and 37 age- and IQ-matched control participants. Participants performed an incentive delay task that allows the investigation of neural activity associated with the anticipation and receipt of monetary and social rewards.

**Results:**

Nonsignificant tests suggested that oxytocin did not influence neural processes related to the anticipation of social or monetary rewards in either group. Complementary Bayesian analyses indicated moderate evidence for a null model, relative to an alternative model. Our results were inconclusive regarding possible oxytocin effects on amygdala responsiveness to social rewards during reward consumption. There were no significant differences in reward-related brain function between the two groups under placebo.

**Conclusions:**

Our results do not support the hypothesis that intranasal oxytocin generally enhances activation of reward-related neural circuits in men with and without ASD.

Difficulties in social communication and interaction are at the core of autism spectrum disorder (ASD). For example, children with autism show low levels of eye contact (1), rarely point at objects to initiate joint attention (2,3), and may struggle to build and maintain friendships (4). It has been hypothesized that these difficulties relate to a diminished sensitivity to social rewards, such as smiles, praise, or gestures of approval, which leads to a lack of orienting toward social stimuli (5, 6, 7). While behavioral and psychophysiological studies seem to support this notion and often highlight diminished responsiveness particularly to social rewards in individuals with ASD (8, 9, 10, 11, 12), neuroimaging studies mostly indicate reduced responses to both social and nonsocial rewards (e.g., money) (13,14). Although these studies have yielded mixed results, meta-analytic findings suggest that on average, individuals with ASD show less pronounced activation in the caudate nucleus, the nucleus accumbens located in the ventral striatum, and the anterior cingulate cortex across different reward tasks (14). These regions are considered key structures within the reward circuit (15, 16, 17). This complex circuit consists of several striatal, cortical, and midbrain regions as well as amygdala, hippocampus, and specific brainstem structures and is thought to be a central component underlying the development and control of motivated behaviors (15, 16, 17).

In the last 15 years, the hormone and neuropeptide oxytocin has gained attention as a modulator of social cognition and behavior (18, 19, 20, 21, 22). Importantly, studies in mice have demonstrated that oxytocin plays a crucial role in the processing of social rewards through coordinated activity with serotonin in the nucleus accumbens (23) and by regulating dopamine release in midbrain structures during social interactions (24,25). In humans, results from functional brain imaging studies suggest that intranasally administered oxytocin can influence neural activity of regions within the reward circuit, such as the anterior cingulate cortex (26), nucleus accumbens (27), and midbrain regions (28,29). Further evidence for a role of oxytocin in reward processing is provided by investigations of oxytocin receptor distribution in the human brain showing that oxytocin receptors are located in regions of the reward circuit, such as the amygdala, anterior cingulate cortex, and brainstem areas, among others (30).

With oxytocin being involved in a range of social behaviors (31), it has been suggested that variation in the oxytocin system contributes to the etiology of ASD (32). A growing body of literature indicates associations of blood oxytocin levels (33, 34, 35, 36, 37, 38) and oxytocin pathway genes with the degree of social difficulties or the diagnosis of ASD per se (39, 40, 41). This evidence has led to a rising interest in intranasal oxytocin as a potential treatment of social symptoms in ASD. Several clinical trials have investigated the effects of a single or repeated doses of oxytocin, with overall inconclusive results to date (42,43). However, clinical studies using functional magnetic resonance imaging (fMRI) suggest that oxytocin may influence regions within the reward circuit in individuals with autism.

Two of these studies explicitly examined oxytocin effects on reward processing and yielded contradictory results. One study testing men with ASD reported that intranasal oxytocin specifically increased learning from social cues and social reinforcement, which was accompanied by a stronger association of reward prediction errors and nucleus accumbens activity compared with placebo (44). In contrast, another study in children with ASD found enhanced activity within regions of the reward circuit only during the anticipation of nonsocial rewards after oxytocin administration (45). Overall, little is known about the influence of oxytocin on the processing of social versus nonsocial rewards in individuals with ASD. Important questions, such as whether this potential influence is similar or different in individuals without ASD and which experimental conditions (e.g., reinforcement learning vs. reward anticipation) might contribute to its efficacy, remain to be answered.

To address these questions, we conducted a randomized, double-blind, placebo-controlled, crossover fMRI study examining the effect of a single dose of oxytocin on reward-related brain function in 37 men with ASD and 37 age- and IQ-matched control participants. Participants performed a well-established incentive delay task that allows the investigation of neural activity associated with the anticipation and receipt of monetary and social rewards (46, 47, 48). The incentive delay task was embedded in a multicenter trial with the overall goal to compare oxytocin effects on neural activation across different facets of social cognition and affect in men with and without ASD (49,50).

## Methods and Materials

### Participants

Participants were enrolled at the University of Lübeck/University Hospital Schleswig-Holstein in Lübeck, Germany, and the Max Planck Institute for Human Cognitive and Brain Sciences in Leipzig, Germany, between December 2016 and January 2019. The sample consisted of 37 men with a confirmed ICD-10 diagnosis of Asperger’s syndrome (*n* = 28), infantile autistic disorder (*n* = 6), or atypical autism (*n* = 3) and 37 healthy male control participants were matched one-on-one for age (±7 years) and Full Scale IQ [±7 IQ points on the Wechsler Adult Intelligence Scale (51)] (Table 1). Eligible participants were German native speakers between 19 and 40 years of age without intellectual impairments (IQ >70). For details on participant recruitment and eligibility criteria, see Supplemental Methods. This study was approved by the Ethics Committee of the University of Lübeck, Lübeck, Germany, and the German Federal Institute for Drugs and Medical Devices (Bundesinstitut für Arzneimittel und Medizinprodukte), Bonn, Germany, and was carried out in compliance with the Declaration of Helsinki. Full informed written consent was obtained from all participants. Of the enrolled 74 participants, one control participant discontinued the study after the first MRI session, leaving data from 73 participants to be analyzed (Figures S1 and S2).

**Table 1:** Sample Characteristics

	ASD (*n* = 37)		Control (*n* = 36)	Cohen’s *d*	95% Confidence Interval	
	*n*	Mean ± SD	Mean ± SD		Lower	Upper
Comorbidities						
ADHD	1					
SDD-MF	2					
DWE	1					
ADOS Total^a^		11.5 ± 4.5				
ADOS SA^b^		11.6 ± 4.6				
ADOS RRB^c^		2.1 ± 2.5				
Age		26.2 ± 4.7	27.1 ± 4.4	−0.20	−0.66	0.26
BMI		24.3 ± 3.8	23.2 ± 2.7	0.33	−0.14	0.80
Full Scale IQ (WAIS IV)		106.9 ± 14.2	109.3 ± 12.3	−0.19	−0.65	0.28
Verbal IQ (WST)		110.2 ± 13.8	112.6 ± 12.3	−0.19	−0.65	0.28
AQ		30.3 ± 9.2	15.0 ± 4.8	2.08	1.41	2.73
IRI Empathy Score		60.5 ± 15.1	72.1 ± 9.1	−0.92	−1.43	−0.40
TAS-20		47.6 ± 12.1	38.0 ± 8.3	0.92	0.41	1.42
BVAQ		116.0 ± 23.2	113.0 ± 19.0	0.14	−0.33	0.61
BDI-II		5.5 ± 5.2	4.0 ± 4.8	0.30	−0.17	0.76
SIAS		33.4 ± 16.3	20.4 ± 12.0	0.90	0.39	1.40
STAI-T		43.8 ± 10.4	35.0 ± 8.6	0.92	0.41	1.43

Information on comorbidities was provided by the referring clinics and university centers. Comorbidities were not additionally assessed or validated at the study centers.

ADHD, attention-deficit/hyperactivity disorder; ADOS, Autism Diagnostic Observation Schedule; ADOS RRB, ADOS repetitive and restricted behavior score; ADOS SA, ADOS social affect score; AQ, Autism Spectrum Quotient; ASD, autism spectrum disorder; BDI-II, Beck Depression Inventory; BMI, body mass index; BVAQ, Bermond-Vorst Alexithymia Questionnaire; DWE, disorder of written expression; IRI, Interpersonal Reactivity Index, empathy score (sum score excluding personal distress subscale); SDD-MF, specific developmental disorder of motor function; SIAS, Social Interaction Anxiety Scale; STAI-T, State-Trait Anxiety Inventory–trait; WAIS IV, Wechsler Adult Intelligence Scale IV; TAS-20, Twenty-Item Toronto Alexithymia Scale; WST, Wortschatztest.

a Data available from 34 participants.

b Data available from 31 participants.

c Data available from 21 participants.

### Trial Design and Procedure

This clinical trial was registered in the German Clinical Trials Register (registration number: DRKS00010053) on April 8, 2016. This multicenter trial used a randomized, double-blind, placebo-controlled, crossover protocol. After a screening procedure, eligible participants were invited to three visits. During the first visit, all participants received detailed information about the study and gave written consent. Participants then filled out several health- and personality-related questionnaires (for details, see Supplemental Methods). Participants with ASD were then randomly allocated to a treatment arm determining the order of treatment (oxytocin first/placebo first) using computer-generated randomization lists. Control participants were assigned to the same treatment arms as the participants they were matched to. Details on the screening process, randomization, blinding, and additional measures during study visits are provided in the Supplemental Methods.

During the second and third visits, participants self-administered 24 IU of the nasal spray (6 puffs per nostril, each containing 2 IU) over the course of several minutes under the guidance of an investigator. Approximately 40 minutes after substance administration, participants entered the scanner and performed three independent experiments, one of which was the reward experiment (Supplemental Methods, Table S1). Results from the other two experiments will be reported separately. Except for the administered nasal spray, the second and third visits included identical procedures and measurements. The visits were 14 days apart and conducted at the same time of day whenever possible (Figure S4).

### Task and Stimuli

**Figure 1:**
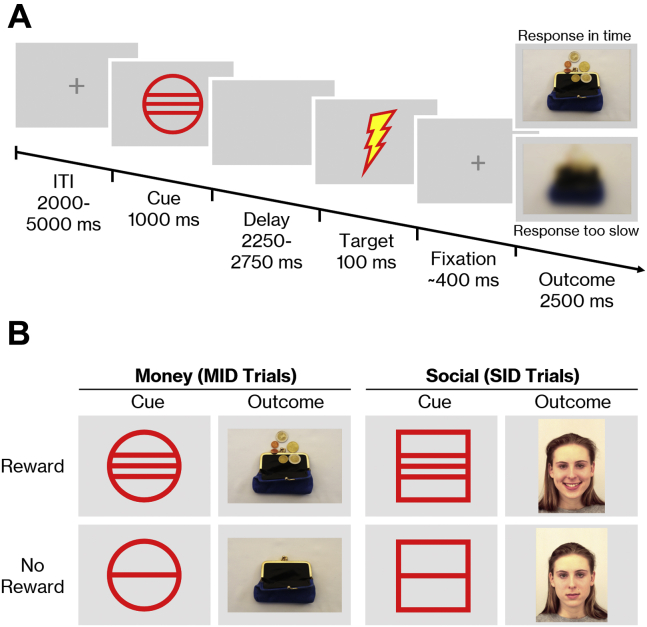
The monetary incentive delay (MID) and social incentive delay (SID) paradigm. **(A)** Timing of the task. Participants were asked to hit a button as fast as possible when the target appeared on the screen. Sufficiently fast responses were followed by an image of either a face or a wallet. Blurred images were shown when responses were too slow. Task difficulty was standardized to a hit rate of approximately 66% by adjusting the time window for responses to individual response times. A first estimate of individual response times was calculated during a training phase at the beginning of the experiment, and the time window was continuously adjusted to the participant’s performance during the main experiment. The experiment was run using Presentation software. Stimuli were presented on a screen positioned behind the magnetic resonance imaging scanner, which participants viewed through a mirror mounted to the head coil. Participants responded to the target by pressing a button on a fiberoptic response box with the index finger of their right hand. **(B)** Social and monetary outcome stimuli and associated cues. To create a reward anticipation phase, a cue indicating the outcome for sufficiently fast responses was presented before the target. Circle cues signaled pictures of wallets, while squares signaled pictures of faces. Horizontal lines within the circles and squares further indicated whether or not the picture would contain a reward: three lines signaled a smiling face (social reward) or a wallet filled with coins (monetary reward); a single line signaled a face with a neutral expression or an empty wallet (no reward). For the face stimuli, 26 color photographs displaying 2 different expressions of 13 people (7 female, 6 male) were taken from the Karolinska Directed Emotional Faces database (77) (stimuli shown here: AF05HAS, AF05NES). The money stimuli consisted of 28 self-created color photographs displaying 14 different wallets, each once empty and once filled with coins.

A monetary incentive delay task (48) and social incentive delay task (46,47) were used to examine neural responses of participants during the anticipation and consumption of rewards. Thirty-six monetary incentive delay task trials and 36 social incentive delay task trials were mixed and presented in a pseudorandomized order. Participants were required to press a button whenever a target symbol appeared on the screen. Sufficiently fast responses (hit trials) were followed by a picture of either a face (social incentive delay task) or a wallet (monetary incentive delay task). Blurred pictures were shown when the reaction was too slow (miss trials) (Figure 1A). The proportion of hit trials was kept constant at approximately 66% for each participant by continuous adjustments of the time window for valid responses to the individual reaction times during the task (Table S2). Cues preceding the target indicated which type of picture was going to be presented after a sufficiently fast response (Figure 1B). Before entering the scanner, participants received detailed instructions and were encouraged to respond as fast as possible to all cue types. A test run of 5 trials ensured that the instructions were understood correctly. The total experiment lasted about 15 minutes.

### fMRI Data Acquisition and Analysis

A 3T MRI scanner was used to acquire an anatomical image and a functional image sequence per subject and session. MRI data from 35 patients and 36 control participants were analyzed using SPM12 (52) in MATLAB R2019b (53). For details on image acquisition, preprocessing, motion correction, and first-level analyses, see Supplemental Methods.

Statistical analyses were performed in a two-level, mixed-effects procedure. The first-level general linear model (GLM) for each subject and session included 10 regressors of interest defining the 4 task conditions during the anticipation phase, the 4 task conditions during the outcome phase of successful trials (hits), and the onsets and durations of trials of the 2 task types (money vs. social) during the outcome phase of unsuccessful trials (misses). Additionally, the 6 realignment parameters and their first derivatives were included as regressors to account for motion-related noise.

On the second level, we created separate GLMs for each of the following first-level contrast images: reward > no reward, social > money, and reward intensity × task type, for both the anticipation phase and the outcome phase (hits only), respectively. Each model contained treatment as within-subject factor and group as between-subject factor as well as site, treatment arm, and the order of the reward task within the three subexperiments as covariates. First, we examined the average task effect across groups and treatments. Second, we compared task-related brain activation of the ASD and control groups under placebo. Third, we examined effects of oxytocin treatment across both groups. Fourth, we examined interaction effects of group and treatment.

To increase the sensitivity of our analyses, we first restricted the search space to a priori defined regions of interest (ROIs). These consisted of the ventral striatum for the anticipation phase and the amygdala for the outcome phase (for details, see Supplemental Methods). Bayes factor repeated measures GLMs with default prior scales (54,55) were conducted to provide relative evidence for or against treatment and group effects on brain activation. In line with Lee and Wagenmakers (56), we describe Bayes factors between 1 and 3 as anecdotal evidence, Bayes factors between 3 and 10 as moderate evidence, and Bayes factors greater than 10 as strong evidence. In a next step, all contrasts of interest were also explored in the whole brain. Results are reported at *p* < .05, familywise error (FWE)–corrected at the voxel level for multiple comparisons within the respective ROI or the whole brain. For further exploratory analyses, see Supplemental Methods.

### Behavioral Data Analysis

Response times were analyzed using a repeated-measures GLM with task type (money vs. social), reward intensity (reward vs. no reward), and treatment (oxytocin vs. placebo) as within-subject factors and group (ASD vs. control) as between-subject factor. Further, site (Leipzig vs. Lübeck), treatment arm (oxytocin first vs. placebo first), and order of the reward task within the three subexperiments (1, 2, or 3) were included as between-subject factors. Further, response times were examined using a Bayesian repeated-measures GLM with default prior scales and identical predictors (Supplemental Methods).

## Results

### Behavioral Data

**Figure 2:**
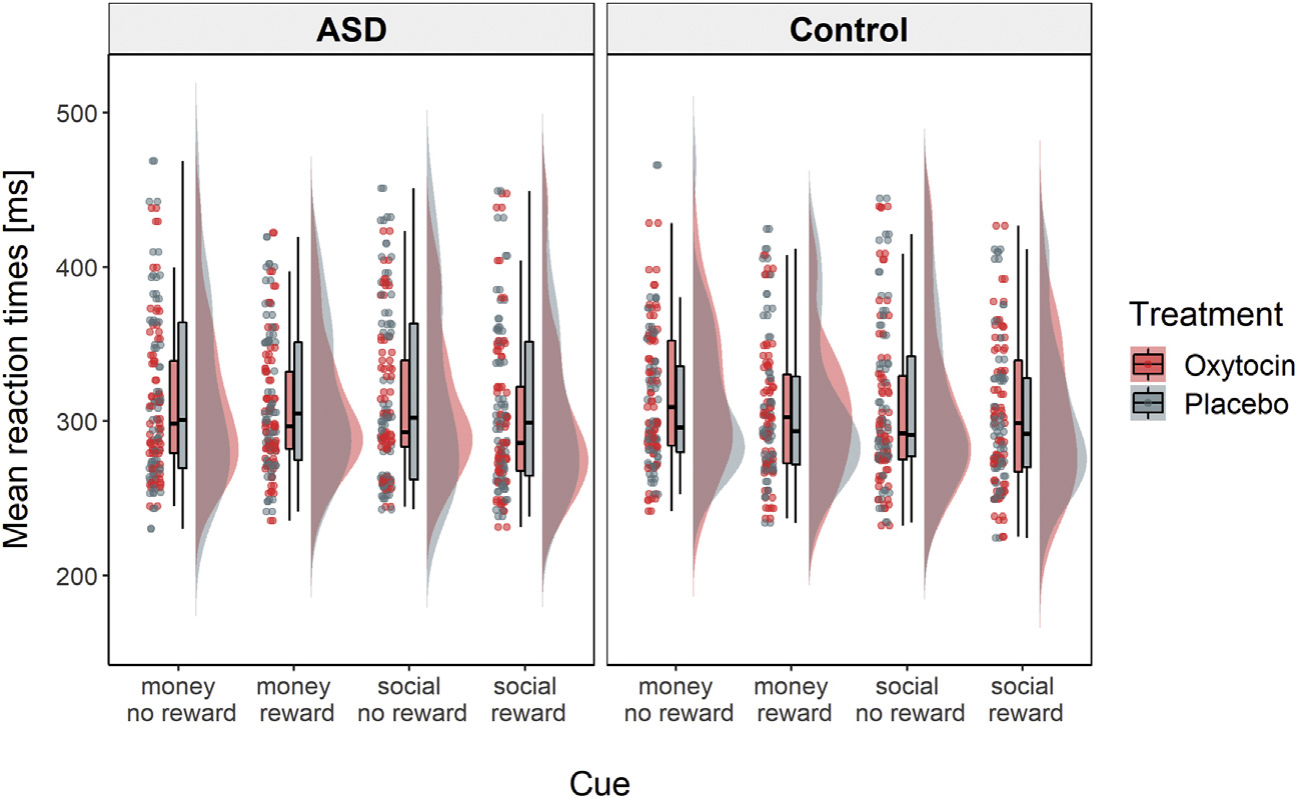
Mean response times for each task type and level of reward intensity. On average, response times were faster when participants anticipated reward compared with no reward. There were no significant differences between the autism spectrum disorder (ASD) group and control group and no effects of treatment on mean response times.

A repeated-measures GLM revealed a significant main effect of reward intensity on mean response times (*F*_1,67_ = 20.61, *p* < .001, η^2^_p_ = 0.235), with faster responses during the anticipation of reward (mean [SD] = 307 [43.6] ms) compared with no reward (mean [SD] = 315 [48.4] ms). There were no significant main effects of group or treatment and no significant interaction effects (Figure 2; Table S3). A Bayes factor repeated-measures GLM yielded very strong evidence for the main effect model of reward intensity compared with the null model (Bayes factor 10 = 69.84). The estimated inclusion Bayes factor for the effect of reward intensity suggested that the data were 79.07:1 in favor of models including this effect compared with models without this effect (Table S3). For all other effects, the inclusion Bayes factors suggested that the data were more likely to occur under models without them (Table S3).

### Imaging Data

#### Anticipation Phase

**Figure 3:**
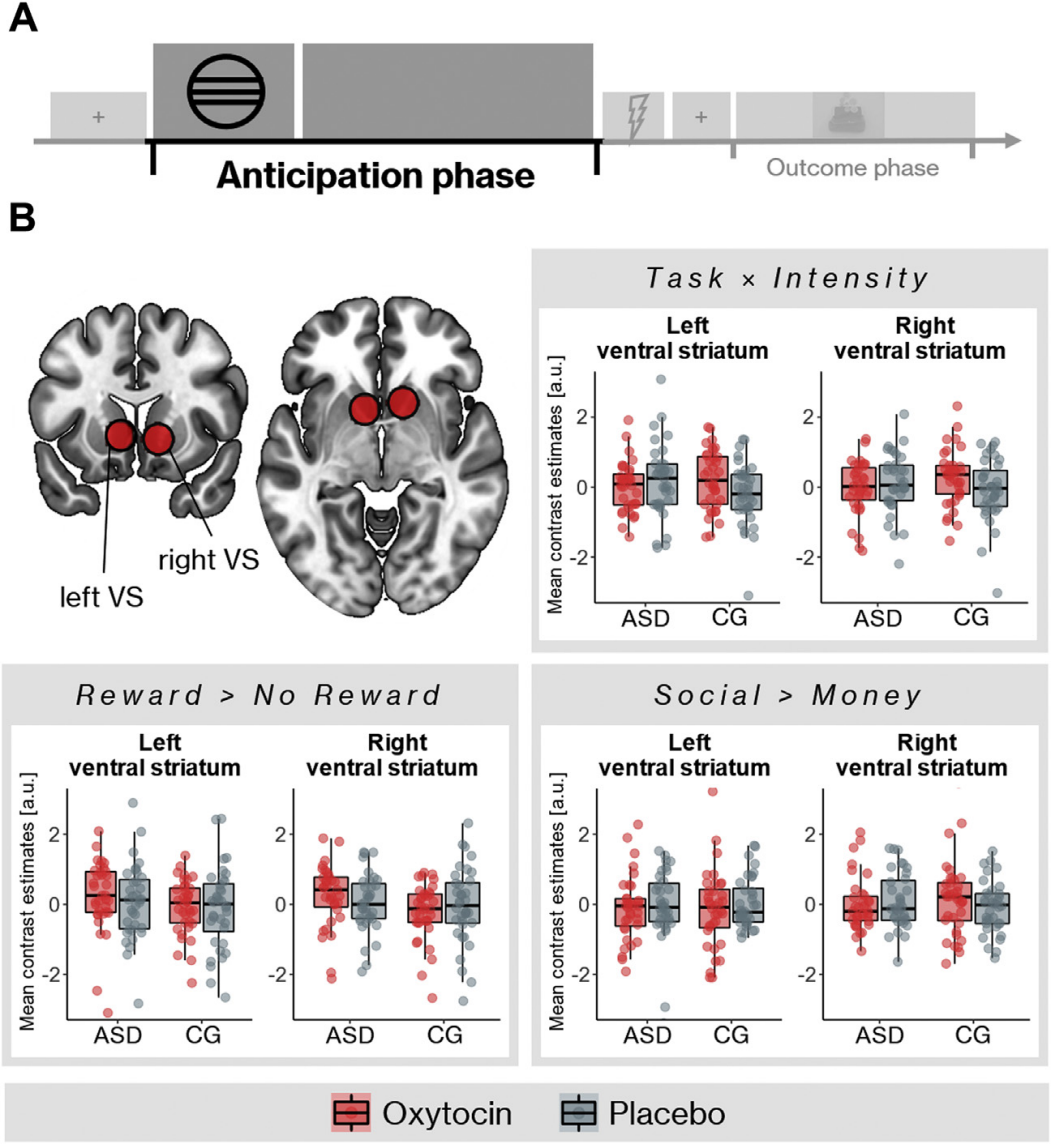
Ventral striatum (VS) activation during the reward anticipation phase. **(A)** The anticipation phase was defined as the time interval within each trial between the start of the cue presentation and the target. **(B)** Regions of interest for the VS (top left panel) and mean contrast estimates for autism spectrum disorder (ASD) group and control group (CG). Contrast estimates for each contrast of interest were extracted from 8-mm spheres around peak coordinates (Montreal Neurological Institute coordinates: left, −10, 10, −2; right, 12, 14, −4) from a meta-analysis examining reward anticipation in the VS (78). There were no statistically significant differences between patients and control subjects and no significant effects of treatment on task-related activation.

All cues induced significant ventral striatum activation compared with baseline (Figure S6; see Table S4 for correlations between response times and ventral striatum activation). On average, difference contrasts of interest (social > money, reward > no reward, task × intensity) were not associated with ventral striatum activation (Figure 3). There were no significant differences between the two groups in the placebo condition for all contrasts of interest. Treatment did not significantly influence task-related activation in the ventral striatum, and there were no significant group × treatment interactions (Figure 3). Analyses of mean extracted data from the ventral striatum confirmed these results. Bayesian repeated-measures GLMs yielded mostly moderate evidence for the null model compared with every model of interest (Table 2). There were no significant group differences, effects of treatment, or group × treatment interactions on whole-brain activation, even when using a more liberal threshold of *p* < .001 and clusterwise FWE control (see Table S5 for task-related whole-brain activation).

**Table 2:** Treatment and Group Effects on Ventral Striatum Activation During Reward Anticipation

Contrast of Interest	Effect/Model	Left Ventral Striatum				Right Ventral Striatum			
		NHST		Bayes		NHST		Bayes	
		*p*	η^2^_p_	BF_10_	BF_incl_	*p*	η^2^_p_	BF_10_	BF_incl_
Reward > No Reward	Group	.199	0.024	0.453	0.453	.084	0.043	0.661	0.661
	Treatment	.481	0.007	0.226	0.226	.897	< 0.001	0.191	0.191
	Group × treatment	.681	0.002	0.030	0.295	.215	0.022	0.063	0.53
Social > Money	Group	.872	< 0.001	0.208	0.208	.12	0.035	0.218	0.218
	Treatment	.446	0.008	0.256	0.256	.606	0.004	0.260	0.260
	Group × treatment	.733	0.002	0.013	0.256	.138	0.032	0.018	0.046
Task × Intensity	Group	.471	0.008	0.236	0.236	.689	0.002	0.212	0.212
	Treatment	.478	0.007	0.249	0.249	.493	0.007	0.239	0.239
	Group × treatment	.095	0.04	0.082	1.448	.101	0.039	0.051	1.131

The analyses were conducted on mean extracted contrast estimates from the left and right ventral striatum, which were corrected for site, arm, and order of the reward experiment within the three subexperiments. The BF_10_ column shows the relative evidence for the model of interest compared with the null model. A BF_10_ >1 indicates that the data are more likely under the model of interest, and a BF_10_ <1 indicates that the data are more likely under the null model. The BF_incl_ column shows the BF_incl_ across matched models as implemented in JASP 0.14.1 (81). This procedure compares models that contain the effect to equivalent models stripped of the effect. For main effect models (i.e., group and treatment), this corresponds to BF_10_. For the models containing the group × treatment interaction, BF_incl_ represents the relative evidence of the interaction model, which also includes the two main effects, compared with the model with only the two main effects, thus the evidence for the interaction effect on its own.

BF_10_, Bayes factor 10; BF_incl_, inclusion Bayes factor; NHST, null-hypothesis significance testing.

#### Outcome Phase

**Figure 4:**
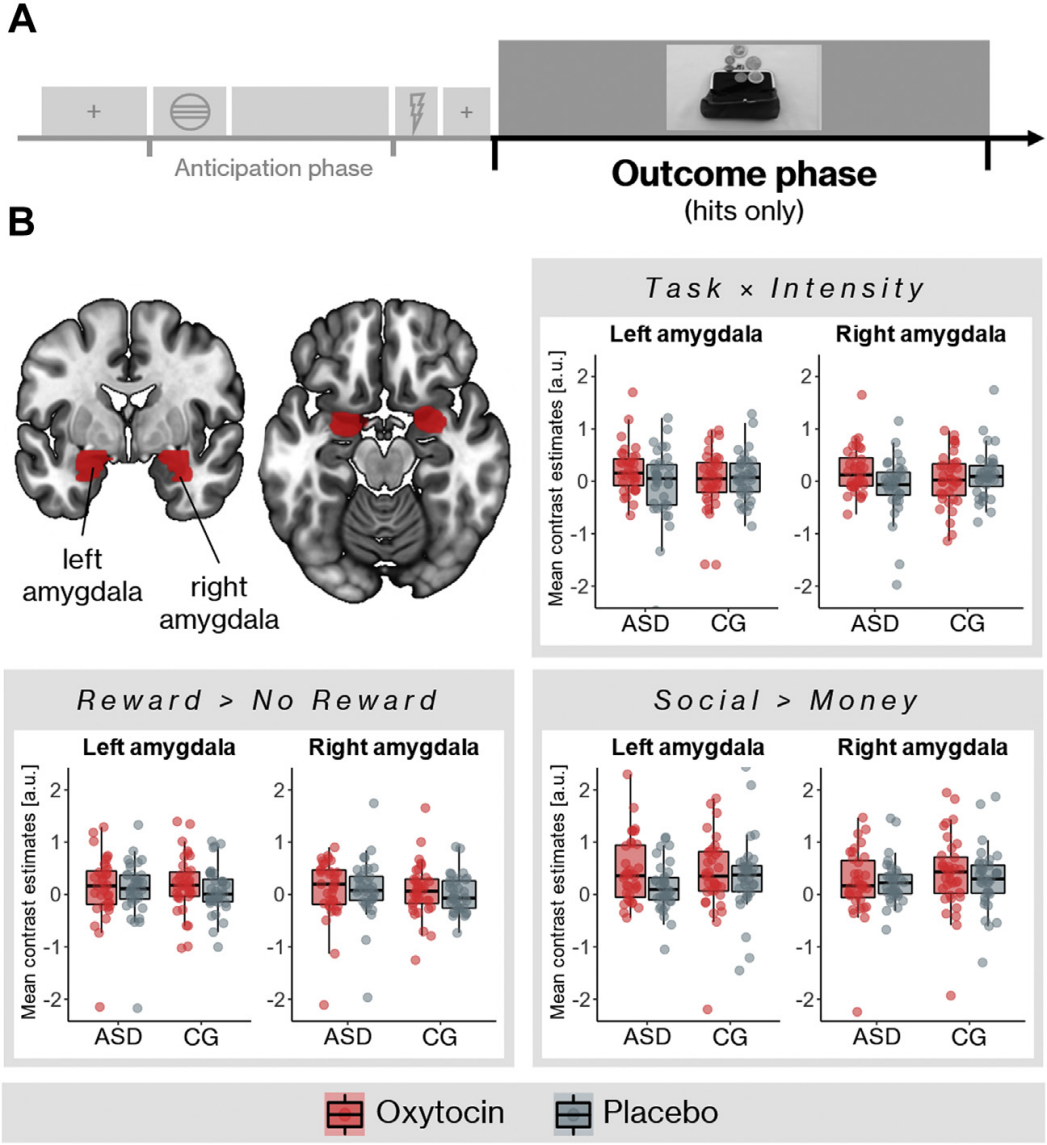
Task-related amygdala activation during the outcome phase of hit trials. **(A)** The outcome phase within a trial. We analyzed hit trials only, i.e., trials with sufficiently fast responses. **(B)** Regions of interest for the amygdala (top left panel) and mean contrast estimates for autism spectrum disorder (ASD) group and control group (CG). A bilateral anatomical amygdala mask was created using the automated anatomic labeling atlas (AAL) (79) integrated in the WFU PickAtlas (80) (dilation factor one). There were no statistically significant differences between patients and control subjects.

On average, the contrasts reward > no reward and social > money were associated with increased activation of the amygdala during hit trials. There were no significant differences between the two groups in the placebo condition for all contrasts of interest (Figure 4). Treatment had a significant effect on the interaction of task and intensity in a single voxel within the left amygdala (Montreal Neurological Institute coordinates: −21, −4, −25, *p*_FWE_ = .031): compared with placebo, oxytocin led to an increase in differential reward sensitivity for social compared with monetary rewards. There were no significant group × treatment interactions on task-related amygdala responses.

Analyses of mean extracted data partly confirmed these results (Table 3). There was a significant group × treatment interaction on mean contrast estimates of task × intensity in the right amygdala (*F*_1,69_ = 4.84, *p* = .031, η^2^_p_ = 0.065) but no other significant effects. In post hoc analyses, we resolved this interaction by calculating the difference between contrast estimates for the oxytocin and the placebo sessions for every participant, thus modeling the increase in social reward sensitivity after oxytocin. This effect was more pronounced in the ASD group (*t*_69_ = 0.031, Cohen’s *d* = 0.52, mean difference: −0.085; Bayes factor 10 = 1.87). Bayesian repeated-measures GLMs showed that the data were always in favor of the null model but yielded only anecdotal evidence against some effects (Table 3).

**Table 3:** Treatment and Group Effects on Amygdala Activation During Reward Consumption

Contrast of Interest	Effect/Model	Left Amygdala				Right Amygdala			
		NHST		Bayes		NHST		Bayes	
		*p*	η^2^_p_	BF_10_	BF_incl_	*p*	η^2^_p_	BF_10_	BF_incl_
Reward > No Reward	Group	.8	< 0.001	0.218	0.218	.449	0.008	0.275	0.275
	Treatment	.379	0.011	0.267	0.267	.657	0.003	0.193	0.193
	Group × treatment	.652	0.003	0.016	0.287	.65	0.003	0.014	0.271
Social > Money	Group	.254	0.019	0.374	0.374	.262	0.018	0.456	0.456
	Treatment	.211	0.023	0.387	0.387	.616	0.004	0.204	0.204
	Group × treatment	.701	0.002	0.038	0.266	.501	0.007	0.022	0.288
Task × Intensity	Group	.714	0.002	0.226	0.226	.69	0.002	0.221	0.221
	Treatment	.207	0.023	0.380	0.380	.23	0.021	0.384	0.384
	Group × treatment	.059	0.051	0.116	1.297	.031	0.065	0.245	3.017

The analyses were conducted on mean extracted contrast estimates from the left and right amygdala, which were corrected for site, arm, and order of the reward experiment within the three subexperiments. The BF_10_ column shows the relative evidence for the model of interest compared with the null model. A BF_10_ >1 indicates that the data are more likely under the model of interest, and a BF_10_ <1 indicates that the data are more likely under the null model. The BF_incl_ column shows the BF_incl_ across matched models as implemented in JASP 0.14.1 (81). This procedure compares models that contain the effect to equivalent models stripped of the effect. For main effect models (i.e., group and treatment), this corresponds to BF_10_. For the models containing the group × treatment interaction, BF_incl_ represents the relative evidence of the interaction model, which also includes the two main effects, compared with the model with only the two main effects, thus the evidence for the interaction effect on its own.

BF_10_, Bayes factor 10; BF_incl_, inclusion Bayes factor; NHST, null-hypothesis significance testing.

There were no significant group differences, effects of treatment, or group × treatment interactions on whole-brain activation, even when using a more liberal threshold of *p* < .001 and clusterwise FWE control (see Table S6 for task-related whole-brain activation).

## Discussion

The aim of this clinical trial was to examine acute effects of intranasal oxytocin on reward-related brain function in male participants with ASD compared with healthy control participants. Using a well-established fMRI task, we did not find support for the notion that oxytocin substantially influences brain activation related to reward anticipation, neither in ASD nor in control participants. Results from Bayesian analyses complemented the results of null-hypothesis significance tests by providing moderate evidence against oxytocin effects. The results were less conclusive with regard to reward consumption, where whole-brain analyses did not show statistically significant oxytocin effects, but ROI analyses yielded mixed evidence. Further, under placebo, there were no statistically significant differences in reward-related brain function between participants with and without ASD.

While the analyses of ventral striatum activation in the anticipation phase revealed relatively clear evidence against oxytocin effects, results from analyses of amygdala activation in the outcome phase were inconclusive. First, we found evidence for an enhancement of left amygdala responsiveness to the presentation of social rewards in the outcome phase in a single voxel. This result suggests a relative increase in sensitivity to the reward value of faces, but not an increase of activation in response to faces per se. This effect was not replicated in analyses of averaged extracted data from the amygdala, although Bayesian analyses provided only weak evidence against the effect. Second, the analysis of extracted data showed a significant interaction of group and treatment on bilateral amygdala responsiveness to social rewards compared with monetary rewards. While Bayesian analyses provided evidence for the interaction effect on its own compared with a main effects model, the data were overall in favor of the null model. Resolving the interaction in line with the hypothesis that oxytocin increases sensitivity specifically to social rewards, we found only anecdotal evidence for the specificity of this effect in the ASD group. In general, the estimated Bayes factors across the analyses of extracted data mostly provided only weak evidence for or against any model, which suggests insensitive data. Overall, the results from our ROI analyses of amygdala activation cannot rule out small increases in social reward sensitivity following oxytocin administration, an effect that might be stronger for men with ASD. This suggests that oxytocin effects on amygdala responsiveness might depend on the perceived reward value of the face. This is not consistent with previous findings, which, despite some heterogeneity, mostly showed an influence of oxytocin in response to faces per se with apparently diverging effects in men with and without ASD (57, 58, 59, 60). While studies in men with ASD often reported an increase of amygdala activation (57,58), studies in men without ASD mostly demonstrated an attenuation of amygdala activation after oxytocin administration, which has been interpreted as a neural mechanism underlying anxiolytic effects of oxytocin (21,59, 60, 61, 62). However, as our results do not provide clear evidence for or against oxytocin effects, they should be interpreted with caution.

Overall, the results from the present study are in line with current meta-analytic evidence suggesting that, on average, intranasal oxytocin has only small and sometimes nonsignificant effects on social cognition and social functioning in individuals with ASD (42,63). This evidence is further supported by a large-scale clinical trial showing that daily administration of oxytocin did not significantly influence social behaviors in 106 men with ASD, although secondary outcome analyses yielded effects on repetitive behavior and social gaze (64). However, meta-analyses summarize a wide range of heterogeneous tasks, which might mask context-specific effects of intranasal oxytocin (22,65). Yet, our findings conflict with results from previous neuroimaging studies highlighting the potential of oxytocin to influence regions within the reward circuit (26, 27, 28, 29,44,45).

The predominant absence of statistically significant oxytocin effects on brain function, especially during reward anticipation, could be due to several factors. One possibility is that intranasal oxytocin effects depend on specifics of the applied reward task, which could explain the heterogeneity of results from oxytocin trials examining reward-related brain activation in ASD. Even when using the same task, there might be differences between studies that contribute to different results. For example, the incentive delay task is one of the most often used tasks to examine reward processing (66), but several variations exist that use different types of reward stimuli. In our study, smiling faces versus neutral faces and pictures of coins versus empty wallets were chosen as reward stimuli, which differs from the only other trial examining oxytocin effects in autism using an incentive delay task (45). Here, the reward conditions consisted of neutral faces for social reward and a dollar sign for monetary reward, which were contrasted to a “no win” condition, where a blank screen was shown after the participants’ response. Although participants in our study responded faster when they anticipated the reward condition, thus clearly distinguishing between the reward and no reward conditions, the conditions might have been too similar compared with conditions used in the other study. This might also explain why the difference contrast between these conditions was not associated with ventral striatum activation during reward anticipation, although each condition on its own was correlated with ventral striatum activation. Possibly, this similarity in the perceived value of the rewards and subjective relevance of the task might have contributed to different oxytocin effects between studies, as it has been discussed that oxytocin modulates stimulus processing depending on personal relevance of the stimuli (67). However, owing to the current scarcity of studies investigating oxytocin effects on reward processing in ASD, we can only speculate about the influence of experimental designs.

Another reason for the predominant absence of oxytocin effects might be related to an overestimation of effect sizes based on early oxytocin trials. Only a few years ago, oxytocin seemed to be a promising treatment option for ASD, with an estimated combined effect size of Cohen’s *d* = 0.57 (68). Subsequent studies examining the effects of intranasal oxytocin on social cognition and behavior have, however, produced inconsistent results, and some early studies could not be replicated (69, 70, 71). Recent research indicates that the median effect size across human oxytocin studies is 0.14, and most studies examining intranasal oxytocin interventions do not have sufficiently large samples to reliably detect effects of this magnitude (72). Although our sample is considerably larger than the average sample size in oxytocin studies in neurodevelopmental disorders (63), and previous studies on reward-related brain function have shown oxytocin effects even in samples of 15 (44) and 28 (45) participants, this suggests that the problem of data insensitivity might also be present in our sample. The results of Bayesian analyses in our study imply that this is especially the case for potential oxytocin effects on amygdala activation.

In addition to the mostly nonsignificant effects of treatment, we did not find significant differences between the two groups under placebo, neither during the anticipation nor the consumption of social and monetary rewards. This result stands in contrast to a hypothesized social “wanting” dysfunction underlying autistic symptoms (6), which posits atypical reward processing specifically during the anticipation of social rewards. This supposed deficit is the focus of accounts that portray autism as an “extreme case of diminished social motivation” (73) based on findings from behavioral and neuroimaging studies. A closer look at studies that explicitly examine social reward anticipation (wanting) using the incentive delay task, however, gives a less clear impression. Of the three studies so far, only one reported hypoactivation of the ventral striatum during the anticipation of social rewards in participants with ASD (74). Results from this study, however, might not be directly comparable to ours, as their task involved negative social reinforcement (i.e., avoiding angry faces), while we used positive social reinforcement (i.e., presenting smiling faces). The two other studies using a task that also examined positive social reinforcement did not show any significant differences in social wanting (75,76). In this respect, our findings are consistent with results from studies with similar tasks that contradict the social motivation hypothesis of autism, but they also contradict meta-analytic evidence suggesting generally atypical reward processing in ASD (14). It should be noted, however, that the results from Bayesian analyses indicated that some of the nonsignificant group effects in our study might have been due to insensitive data. This was specifically the case for the reward > no reward contrast during the anticipation phase and the social > money contrast during the outcome phase. Consequently, we cannot rule out small potential group differences in reward-related brain activation in key regions of the reward circuit, although the results do not provide evidence for these differences either.

Overall, our results do not support the hypothesis that intranasal oxytocin generally enhances activation of reward-related neural circuits in men with and without ASD. While our results provide relatively clear evidence against oxytocin effects during reward anticipation, they are inconclusive with regard to amygdala responsiveness during the receipt of social rewards, which might be indicative of insensitive data. In line with recent evidence, the effects of intranasal oxytocin are likely too subtle to fundamentally shape reward circuitry regardless of context. Instead, oxytocin efficacy may be influenced by features of the experimental design. Moreover, our results raise doubts about the hypothesis that social reward processing is universally altered in ASD. The utility of oxytocin in improving social reward processing in ASD is likely limited.

## Supplementary Material

Supplementary Material

Key Resources Table
