# Supplementary material for: Assessment of Reward-Related Brain Function After a Single Dose of Oxytocin in Autism: A Randomized Controlled Trial
Source: Biol Psychiatry Glob Open Sci. 2021 Oct 23;2(2):136–46. doi: 10.1016/j.bpsgos.2021.10.004 (PMC9616329; doi:10.1016/j.bpsgos.2021.10.004)
Supplement: Supplementary Material [file mmc1.pdf]

Supplementary Information for

Assessment of Reward-Related Brain Function After a Single Dose of Oxytocin in Autism: A Randomized Controlled Trial

SUPPLEMENTARY METHODS

Participant recruitment and eligibility criteria

Participants with ASD were recruited from five German university centers offering autism specific diagnosis and counselling, as well as through online advertisements and leaflets. In the university centers, participants with ASD had undergone standardized diagnostic procedures including the Autism Diagnostic Observation Schedule (ADOS (1)) and the Autism Diagnostic Interview-Revised if parental informants were available (ADI-R (2); caregivers were available in 70.3% of cases). Control participants were recruited through in-house databases and from the general population via public notices and online advertisements. Eligible participants were male German native speakers between 19 and 40 years of age without intellectual impairments (IQ > 70). Participants of the ASD group were required to have one of the following ICD-10 diagnoses: Asperger's syndrome (F84.5), infantile autistic disorder (F84.0), or atypical autism (F84.1). Participants with ASD who were not referred directly from an autism specific university center were required to provide a report detailing the diagnostic process of their ASD diagnosis (i.e., whether the diagnosis was based on gold standard diagnostic procedures including ADI-R and ADOS) and a confirmation of their ASD diagnosis based on ICD-10 criteria. Further, all participants were required to give written consent. We excluded participants with an IQ lower than 70, a BMI less than 18 or over 30, those with a frequent use of drugs, alcohol, and nicotine (more than 15 cigarettes per day), those with current suicidal tendencies, contraindications to oxytocin (e.g., cardiac arrhythmia), metal implants or any other MRI contraindications, as well as participants who were not able to give consent. Participants receiving concurrent antidepressant (n = 2) or other medication (n = 2) were required to keep the dosage constant during participation. Additional exclusion criteria for control participants included a history of neurological, endocrinological or psychiatric disorders based on self-report, current psychotherapeutic or psychiatric treatment, current use of psychotropic medication, first- or second-degree relatives with autism spectrum disorders, and a score of 32 or higher on the Autism-Spectrum Quotient (AQ) (3).

Sample size calculations

Details on a priori sample size calculations are given in our published study protocol (4). Briefly, based on meta-analytic effect size estimates available at the time of study planning, we assumed relatively strong effects of oxytocin treatment on neural network activity (d = 0.65). A sample of n = 88 participants with ASD (and n = 88 control participants) would be necessary to reach a desired power of 80% at a significance level of α =

0.001 for two-tailed t-test comparisons of oxytocin effects in a 2×2 cross-over design. Because of difficulties with patient recruitment and resulting significant time delays, the study had to be ended prematurely. In total, n = 24 participants were scanned in Lübeck, and n = 50 participants were scanned in Leipzig (Supplementary Figures S1 and S2).

Randomization and Blinding

Randomization was carried out by the Clinical Trial Center (ZKS) Leipzig independently from the recruitment process and data acquisition. The ZKS created blocked randomization lists separately for each study site with a 1:1 allocation ratio and varying block sizes of 2 or 4. According to these lists, participants were allocated to either arm A (oxytocin at first visit, placebo at second visit) or arm B (placebo at first visit, oxytocin at second visit) and received a site-specific medication number. Additionally, the order of experiments during the fMRI sessions was randomized within each treatment arm using blocked randomization with a block size of 6. Blinding was carried out by an independent pharmacist at the Clinic Pharmacy of Heidelberg University Hospital, who received the list of medication numbers and their corresponding treatment arm from the ZKS Leipzig. Oxytocin (Syntocinon® Nasal Spray) and placebo were filled into nasal spray bottles of identical appearance and labeled with a medication number and the according administration sequence (bottle A for first visit, bottle B for second visit). The placebo nasal spray contained all inactive ingredients of the oxytocin nasal spray. Both nasal sprays were identical in appearance and smell. The two bottles were sealed in non-transparent foil, labeled with the respective medication number, and sent to the respective study sites. After receiving a participant's informed written consent, a study nurse filled in an online form provided by the ZKS asserting that all inclusion and no exclusion criteria were met, and subsequently received the medication number and the order of experiments for the respective participant. The center-specific randomization lists were not accessible to investigators engaged in recruitment, data acquisition (including study nurses, study physicians and research assistants), and data analysis. Consequently, all investigators involved in the conduct of the study as well as all participants were blinded to the order of treatment.

Participant screening

Interested participants were first screened for main inclusion criteria. For interested control participants, this screening consisted of a short telephone interview, as well as the completion of the Wechsler Adult Intelligence Scale (WAIS-IV) (5) at the respective study site. If a control participant was matched with an already randomized participant with ASD, he was invited to the first study visit. For interested participants with ASD, successful telephone screening was directly followed by the first study visit. In preparation for the first visit, participants received a copy of the informed consent to read. During the first visit, a study physician gave detailed information on risks and benefits of the study and answered the participant's questions. Following written consent, a study nurse ensured that all inclusion and no exclusion criteria were met. For this purpose, heart rate and blood pressure were measured, and MRI compatibility was assessed using a questionnaire. A brief standardized interview was conducted to exclude possible alcohol abuse or dependence. Participants

filled out several health and personality related questionnaires, including the Autism Spectrum Quotient (AQ) (3), to exclude control participants with high autistic traits. All participants were instructed to abstain from food, caffeine, and excessive amounts of water 2 hours before the beginning of the next two study visits.

At the beginning of the second and third visits it was ensured that none of the inclusion or exclusion criteria had changed since the last visit.

Overview of experimental paradigms

During the second and third visit, participants performed three independent experiments in the MRI, all of which were designed to capture brain function associated with social cognitive and affective processes. In addition to the incentive delay task, participants also completed an emotional matching task and the EmpaTom (6), a task investigating the neural correlates of empathy and theory of mind. The emotional matching task was based on an established fMRI task for studying emotion processing (7, 8). In this task, participants viewed three images of either faces, social scenes, or non-social scenes of varying valence, and were required to indicate which of the two pictures shown in the bottom half of the screen corresponded to the picture presented in the top half of the screen. The matching task took about 10 minutes to complete. During the EmpaTom, participants viewed short video clips of actors recounting putatively autobiographic episodes of varying emotionality. Participants then reported their own affective response to the stories, the degree of compassion experienced towards the actor, and answered a question about the content of the video. This task took about 25 minutes to complete. More details on these tasks are given in our published study protocol (4). All experiments consisted of two parallel versions that were used during the two study visits. Results from the matching task and the EmpaTom will be reported elsewhere.

Additional measures during study visits

During the second and third visits, heart rate and blood pressure were measured several times to monitor potential cardiovascular effects of oxytocin (see Supplementary Figure S3). Each visit started with two baseline measurements of blood pressure and heart rate, followed by nasal spray administration, after which a third and fourth measurement was conducted. The last timepoint was immediately after completion of the MRI experiments, where blood pressure and heart rate were measured twice again.

Further, participants indicated their current feelings of anxiety using the state scale of the State Trait Anxiety Inventory (STAI-S) (9), which was used as a measure to examine potential anxiolytic effects of oxytocin (see Supplementary Analyses). State anxiety was assessed at two timepoints during these visits: once directly after nasal spray administration, and once again after the MRI measurement.

Further, at the end of each session, participants were asked to guess whether they had received oxytocin or placebo. This was done to check if blinding was successful (see Supplementary Analyses).

Subjective side effects of the nasal spray were assessed by directly asking participants if they had experienced any health-related problems after the second and third visit. Participants were instructed to contact the study team in case of subjectively experienced side effects up to two weeks after substance administration. No side effects were reported.

All participants were also asked to provide a saliva sample for cross-sectional (epi)genetic analyses conducted within the multi-center consortium on ASD (10). The donation of the saliva sample was voluntary and independent of the participation in the MRI study. The sample was collected at the beginning of each MRI session (visits 2 and 3) before nasal spray administration.

Health and personality related questionnaires

Over the course of the study, participants were asked to fill out several questionnaires assessing health and personality traits. Since the study was embedded in a Germany-wide research network on mental disorders, some of the data was collected for cross-sectional studies with a focus on understanding mental health and illness from a multidimensional perspective (11). While only some of the questionnaires are used in the current data analysis, all questionnaires and other psychometric tests will also be used for the analyses of the other functional paradigms of this clinical trial. To keep the workload low for participants at the beginning of the study, the assessment battery was divided into two halves and collected at two time points.

The first time point was during the first study visit. The questionnaires and tests collected during the first visit included a questionnaire about basic sociodemographic information, a handedness questionnaire (12), the 53-item Brief Symptom Inventory (13) assessing clinically relevant psychological symptoms, the Toronto Alexithymia Scale (TAS 20) (14, 15), the Beck Depression Inventory (BDI-II) (16, 17) and the Autism Spectrum Quotient (AQ) (3). Moreover, participants completed the Trail Making Test A & B (18) as a measure of visual attention and task switching. Verbal intelligence was assessed using a short vocabulary test (WST) (19).

Between the second and third visits, participants filled out the second half of the questionnaires, which were provided online. The questionnaires included the WHO Disability Assessment Schedule 2.0 (20), the Childhood Trauma Screener (21), the Behavioral Inhibition and Behavioral Activation Scale (22), the Barratt Impulsiveness Scale (short form BIS-15) (23), the Positive and Negative Affect Schedule (24), the Interpersonal Reactivity Index (IRI) (25, 26), the Social Interaction Anxiety Scale (SIAS) (27), the State-Trait Anxiety Inventory – trait (STAI-T) (9), the Cognitive Emotion Regulation Questionnaire (28) and the Bermond-Vorst Alexithymia Questionnaire (BVAQ) (29).

Of all health and personality related questionnaires collected during the clinical trial, only those related to depression (BDI-II), anxiety (SIAS, STAI-T), autistic traits (AQ), empathy (IRI) and alexithymia (TAS-20, BVAQ) were used for the current data analysis.

Analysis of response times

Behavioral data was analyzed using Jamovi version 1.2.27 (30), unless stated otherwise. Trials with response times below 100 ms and above 2000 ms were excluded. Mean response times were computed for each subject, session, and task condition and log-transformed (31). Log-transformed mean response times were analyzed using a repeated-measures general linear model (GLM) with task type (money vs. social), reward intensity (reward vs. no reward), and treatment (oxytocin vs. placebo) as within-subject factors, and group (ASD vs. controls) as between-subject factor. Further, site (Leipzig vs. Lübeck), treatment arm (oxytocin first vs. placebo first) and order of the reward task within the three sub-experiments (1, 2 or 3) were included as between-subject factors to control for these possible confounds.

Stimulus ratings

To include the subjective reward value of the stimuli in our analyses of behavioral and brain imaging data, we asked all participants to rate the stimuli using an online survey that was sent to them after the last visit. Each stimulus was presented with the question “How rewarding was this picture?” and participants were asked to respond on a scale from 1 (not at all rewarding) to 9 (very strongly rewarding). Unfortunately, of the total 73 participants who took part in the study, only 27 completed the online survey, even after multiple reminders. For this reason, we did not include the ratings in any of our analyses. Mean ratings for the four stimulus categories based on the available data from n = 27 participants are shown in Table S7.

Image acquisition

Participants were scanned using a 3T Siemens MAGNETOM Skyra scanner (Siemens, Erlangen, Germany) at the Center for Brain, Behavior and Metabolism (CBBM) in Lübeck and a 3T Siemens MAGNETOM Skyra fit scanner (Siemens, Erlangen, Germany) at the Max Planck Institute for Human Cognitive and Brain Sciences in Leipzig. An echo planar imaging (EPI) sequence was used for the acquisition of functional volumes (number of slices = 50, voxel size = 3 × 3 × 3 mm^3, FoV = 210 × 210 mm^2, TR = 2000 ms, TE = 27 ms, 90° flip angle). Simultaneous multi-slice imaging (slice acceleration factor = 2) was used for accelerated coverage of the whole brain. A high-resolution anatomical image was acquired for normalization using a T1-weighted 3D MPRAGE sequence (number of slices = 176 (sagittal), voxel size = 1 × 1 × 1 mm3, FoV = 240 × 256 mm^2, TR = 2300 ms, TE Lübeck = 5.49 ms, TE Leipzig = 5.52 ms, 9° flip angle).

Imaging data analysis: preparation, pre-processing and first-level analyses

Because of an unexpected loss of fMRI data from one session, one participant with ASD was excluded from the analyses. Another participant with ASD was excluded due to excessive head motion in one session (mean framewise displacement: 0.97 mm) causing aliasing artifacts in the time series data. The remaining data sets from n = 35 patients and n = 36 control participants were analyzed using SPM12 (32) in MATLAB R2019b (33). Since the length of the experiment varied according to the subjects’ mean response times, the MRI measurements were terminated manually, which led to variance in the total number of images in the

timeseries. The images to be analyzed were defined as all images up to two seconds after the end of the last trial, resulting in 381-383 images per subject and session. Functional volumes were slice-time corrected, spatially realigned, and normalized using the forward deformation fields as obtained from the unified segmentation of the anatomical T1 image. To remove low frequency drifts, the images were high pass filtered at 1/128 Hz.

Statistical analyses were performed in a two-level, mixed-effects procedure. The first-level GLM for each subject and session included ten regressors of interest defining the onsets and durations of the four task conditions during the anticipation phase (social:reward, social:no reward, money:reward, money:no reward), the four task conditions during the outcome phase of successful trials (hits) and the onsets and durations of trials of the two task types (money vs. social) during the outcome phase of unsuccessful trials (misses). Additionally, the six realignment parameters and their first derivatives were included as regressors to account for motion-related noise. Smoothed contrast images were used in the analyses on the second level.

Motion correction

We adopted two measures to mitigate the impact of head-motion related artefacts. First, the six realignment parameters obtained during preprocessing as well as their first derivatives were included as regressors of no interest in the first-level analyses. Second, we used the RobustWLS toolbox (35) to estimate the variance of the noise for each image in the time series and obtain a weighted least squares estimate of the regression parameters. Because this method works optimally on spatially unsmoothed data (35), all first-level analyses were performed with unsmoothed data, and smoothing was applied on the resulting contrast images using an 8 mm full-width half-maximum isotropic Gaussian kernel before they were used in the second-level analyses.

Correction of differences in image acquisition

Due to slightly different hardware and software configurations during image acquisition at the two study sites, the functional images showed differently distributed luminance values. To prevent signal dropout due to these differences, the implicit masking threshold of the linear models on the first level was lowered to 0.3 (default value in SPM 12: 0.8). For whole-brain analyses on the second level, we then used a mask to restrict the analyses to within-brain voxels, since signal from non-brain tissue was sometimes included in the contrast images due to the liberal analysis threshold on the first level. This mask was obtained by calculating average images of normalized grey and white matter tissue maps across all included subjects. The resulting two average images of grey and white matter were summed up to create a single mask. Whole-brain analyses were thresholded at a voxel level of $p < .05$, family-wise error (FWE)-corrected for multiple comparisons within the whole-brain mask. Since voxel-wise FWE-correction is relatively conservative, we also explored whole-brain results using the more liberal cluster-wise FWE-correction at a cluster-forming threshold of $p < .001$.

Region-of-Interest (ROI) analyses

To increase the sensitivity of our analyses, we first restricted the search space to a priori defined regions of interest (ROI). The ventral striatum was chosen as ROI for the anticipation phase due to its pivotal role during reward anticipation (37, 39, 41). The bilateral ventral striatum mask consisted of two 8 mm spheres around peak coordinates (MNI coordinates left: -10, 10, -2; right: 12, 14, -4) from a meta-analysis of ventral striatum activation associated with reward anticipation (34). For the outcome phase, the amygdala was chosen as ROI because of its associations with reward consumption (42) and intranasal oxytocin effects (43, 44). A bilateral anatomical amygdala mask was created using the automated anatomic labeling atlas (AAL) (36) integrated in the WFU PickAtlas (38) (dilation factor one). All contrasts of interest were examined within these masks, using small volume correction to account for the smaller search area. Additionally, to reduce potential noise, we extracted contrast estimates for the contrasts of interest from these ROIs and used the averaged signal as outcome measures in repeated measures GLMs with treatment as within-subject factor and group as between-subject factor.

Bayesian analyses

Non-significant results from frequentist statistical tests do not provide support for the absence an effect. To be able to interpret non-significant results, especially with regards to potential oxytocin effects, we also applied Bayesian statistics in the analyses of behavioral data and ROI-extracted data using JASP version 0.14.1 (40). We calculated Bayesian repeated measures ANOVAs with default prior scales (45, 46) and included the same predictors as in the frequentist ANOVAs. We report the resulting Bayes factors (BF) for the models and effects of interest. The Bayes factor is a ratio comparing the likelihood of the data fitting under the null hypothesis with the likelihood of fitting under the alternative hypothesis, and thus provides relative evidence for or against these hypotheses.

For the analysis of response times, we calculated Inclusion Bayes factors across matched models for all effects of interest as implemented in JASP (40, 47). This was done to quantify the relative evidence for or against certain predictors across several models, rather than studying the results for each model individually. The Inclusion Bayes factor across matched models reflects the likelihood ratio of models that contain the specific predictor to equivalent models stripped of the predictor. For the analysis of extracted imaging data, we report Bayes factors comparing each model to the null model, as well as inclusion Bayes factors for interaction effects.

In JASP version 0.14.1, the default priors for repeated-measures ANOVAs are specified as three zero-centered Cauchy priors with different scales for fixed effects ($r = 0.5$), random effects ($r = 1$), and covariates ($r = 0.354$), respectively (46). To assess the sensitivity of our analyses to the chosen priors, we conducted the same analyses using a wider ($r = 1$) and narrower scale ($r = 0.2$) for the fixed effects in our models (60). Since our models only contained fixed effects and no random effects or covariates, only the hyperparameter for fixed effects was changed. Results of these analyses are presented in Supplementary Tables S11 and S12.

Interactions of treatment and individual difference variables on reward sensitivity

In an exploratory approach, we examined whether interindividual differences in age, IQ, anxiety (27, 48), depression (16, 17), autistic traits (3) empathy (25, 26) and alexithymia (15, 29) influence the magnitude of oxytocin effects on behavioral and neural markers of reward sensitivity. These analyses were conducted using R version 4.0.3 (49). As a measure of behavioral reward sensitivity, we calculated response time differences for reward and no reward trials for social and monetary cues, respectively (money: no reward-reward and social: no reward-reward). This was done separately for the oxytocin and placebo sessions. Next, all variables of interest were correlated with behavioral reward sensitivity under oxytocin and placebo. For each variable of interest, the resulting two correlation coefficients were then tested for differences using Steiger's approach for dependent overlapping correlations (50) as implemented in the cocor R package (51). The results of these correlation analyses are reported in Supplementary Table S8 (see also Supplementary Figure S5 for intercorrelations of the individual difference variables).

As with reaction times, we examined interactions between treatment and differences in age, IQ, anxiety, depression, autistic traits, empathy, and alexithymia on reward-related brain activation. For each phase, we extracted average contrast estimates for money: reward > no reward and social: reward > no reward from the respective ROI. This was done separately for oxytocin and placebo sessions. Next, all individual difference variables were correlated with average contrast estimates under oxytocin and placebo and tested for differences as described above. The results of these correlation analyses are reported in Supplementary Tables S9 and S10.

SUPPLEMENTARY ANALYSES

Oxytocin effects on state anxiety

To examine possible anxiolytic effects of oxytocin, we calculated a repeated-measures GLM with state anxiety (measured with STAI-S) as the dependent variable. Treatment and time (pre-scan vs. post-scan) were entered as within-subject factors, and group, site, and treatment arm (oxytocin first, placebo first) were entered as between-subject factors.

There was a significant main effect of treatment ($F(1,67) = 4.01$, $p = .049$, $\eta_p^2 = .056$) and a significant interaction of treatment and arm ($F(1,67) = 5.78$, $p = .019$, $\eta_p^2 = .079$). Post-hoc t-tests revealed significant differences in STAI-S sum scores only in the group that received placebo first. Here, anxiety was lower after participants received oxytocin (second session, $M = 31.9$, $SD = 5.76$) as compared to placebo (first session, $M = 33.5$, $SD = 7.20$, $t(67) = 3.19$, $p = .013$, corrected for multiple comparisons using Holm's procedure (52)). There was no such effect in the group that received oxytocin first (oxytocin: $M = 33.4$, $SD = 7.57$, placebo: $M = 33.1$, $SD = 8.17$, $p > .05$). Further, there was a main effect of time ($F(1,67) = 5.58$, $p = .021$, $\eta_p^2 = .077$), indicating that participants felt more anxious before MRI scanning than after scanning (pre: $M = 34.0$, $SD = 6.98$; post: $M = 32.6$, $SD = 7.09$). A main effect of group ($F(1,67) = 8.24$, $p = .005$, $\eta_p^2 = .110$) indicated that participants with ASD felt more anxious than control participants (ASD: $M = 35.4$, $SD = 7.46$; control: $M = 31.1$, $SD = 4.97$).

Check for successful blinding

A binomial test was used to determine whether participants were able to identify above chance level which nasal spray they had received. Since some participants indicated the same treatment at both sessions, we compared the proportion of correct guesses per treatment across both sessions to 0.5, instead of analyzing correct guesses per session. For the binomial tests, Bayes factors (BF) are provided in addition to the *p*-values as indicators of relative evidence for and against the alternative hypothesis.

Participants could not identify above chance level which nasal spray they received: 51.5% of participants correctly identified oxytocin ($p = .904$, $BF_{10} = 0.155$) and 53.7% of participants correctly identified placebo nasal spray ($p = .625$, $BF_{10} = 0.182$).

Exploratory analyses of cue-related activation in ventral striatum

To explore whether treatment and group effects were present on a fundamental level and to concentrate on potentially more reliable parameter estimates, mean extracted data from the ventral striatum ROI for all cues against a low-level baseline was examined using a repeated-measures GLM separately for each cue condition. Each GLM contained treatment as within-subject factor and group as between-subject factor. Data from the left and right ventral striatum ROIs were averaged to reduce the number of comparisons.

The analyses yielded a significant main effect of group on ventral striatum responses against baseline in every cue condition (Money – Reward: $F(1,69) = 7.04$, $p = .010$, $\eta_p^2 = .093$, $BF_{incl} = 3.66$; Money – No Reward: $F(1,69) = 6.20$, $p = .015$, $\eta_p^2 = .082$, $BF_{incl} = 2.32$; Social – Reward: $F(1,69) = 6.64$, $p = .012$, $\eta_p^2 = .088$, $BF_{incl} = 3.09$; Social – No Reward: $F(1,69) = 5.78$, $p = .019$, $\eta_p^2 = .077$, $BF_{incl} = 2.01$). There were no significant main effects of treatment, with Bayesian analyses however only providing anecdotal evidence against these effects and thus suggesting data insensitivity (Money – Reward: $F(1,69) = 2.47$, $p = .102$, $BF_{incl} = 0.60$; Money – No Reward: $F(1,69) = 2.93$, $p = .091$, $BF_{incl} = 0.76$; Social – Reward: $F(1,69) = 3.41$, $p = .069$, $BF_{incl} = 0.97$; Social – No Reward: $F(1,69) = 1.83$, $p = .180$, $BF_{incl} = 0.46$). There were no significant treatment × group interactions (Money – Reward: $F(1,69) = 0.01$, $p = .941$, $BF_{incl} = 0.24$; Money – No Reward: $F(1,69) = 0.75$, $p = .390$, $BF_{incl} = 0.34$; Social – Reward: $F(1,69) = 0.39$, $p = .535$, $BF_{incl} = 0.28$; Social – No Reward: $F(1,69) = 0.04$, $p = .838$, $BF_{incl} = 0.26$). Mean parameter estimates for all cue conditions are displayed in Figure S6.

Exploratory functional connectivity analyses

Given more recent evidence showing that intranasal oxytocin may not only alter brain function in specific regions, but may also have broad circuit effects (53–55), we conducted exploratory analyses of task-independent functional connectivity. We applied a seed region approach (56–58) and selected the left and right amygdalae and ventral striatum as seed regions. To determine seed voxels located within the seed regions, we constrained the search space to the masks used in the ROI analyses of task-related neural activation. For the ventral striatum, we identified the next local maximum within each subject for the contrast of all cues vs. baseline during the anticipation phase, starting at the central coordinates of the respective ventral striatum mask (MNI coordinates left: -10, 10, -2, right: 12, 14, -4). For the amygdala, we identified the next local maximum within each subject for the contrast of faces vs. baseline during the outcome phase, starting at -24, -4, -16 for the left amygdala, and 26, -2, -16 for the right amygdala. Seed time series were extracted as the first eigenvariate in a sphere of 6-mm radius as implemented in SPM12. We removed task-related variance by applying an effects-of-interest correction with the F-contrast set on the six movement parameters as well as their first derivatives. To account for noise, two additional time series were extracted similarly for each subject from the first eigenvariates of all voxels within masks covering medial cerebrospinal fluid regions (CSF) or white matter (WM) (58).

The fixed-effects GLMs on the subject-level included the extracted seed time series of the respective seed region, the two WM and CSF noise regressors, and twelve regressors modeling head movement parameters as well as their first derivatives. Ten task-specific regressors (four task conditions during the anticipation phase, the four task conditions during the outcome phase of successful trials and the onsets and durations of trials of the two task types (money vs. social) during the outcome phase of unsuccessful trials) were included to explain variance due to hemodynamic responses induced by the task. For each subject, parameters of the GLM were calculated and the β-maps of the respective seed time series (connectivity maps) were analyzed on the group-level. Here, we assessed treatment and group effects in a random-effects GLM including

treatment as within-subject factor and group as between-subject factor, as well as site, treatment arm and the order of the reward task within the three sub-experiments as covariates of no interest. Whole-brain analyses as well as ROI analyses were performed, using the same ROIs and thresholds as in the analyses of task-related activation differences.

Treatment effects were examined using the contrasts oxytocin > placebo and placebo > oxytocin in the whole brain and in our pre-defined ROIs. There were no significant treatment effects for any of the examined connectivity maps, even when using the more liberal cluster-wise FWE correction and a cluster-defining threshold of $p < .001$. We also examined potential group differences in functional connectivity under the placebo condition. For the functional connectivity of the left ventral striatum, ROI analyses indicated higher functional coupling with the left and right ventral striatum in participants with ASD compared to control participants (left: -9, 11, -4, $t(135) = 3.37$, $p(\text{FWE}) = .010$; right: 12,14, -4, $t(135) = 4.42$, $p(\text{FWE}) = .001$). Other than that, there were no significant group effects, even when using cluster-wise FWE correction. There were no significant group × treatment interaction effects for any of the examined connectivity maps, even when using cluster-wise FWE correction.

SUPPLEMENTARY FIGURES

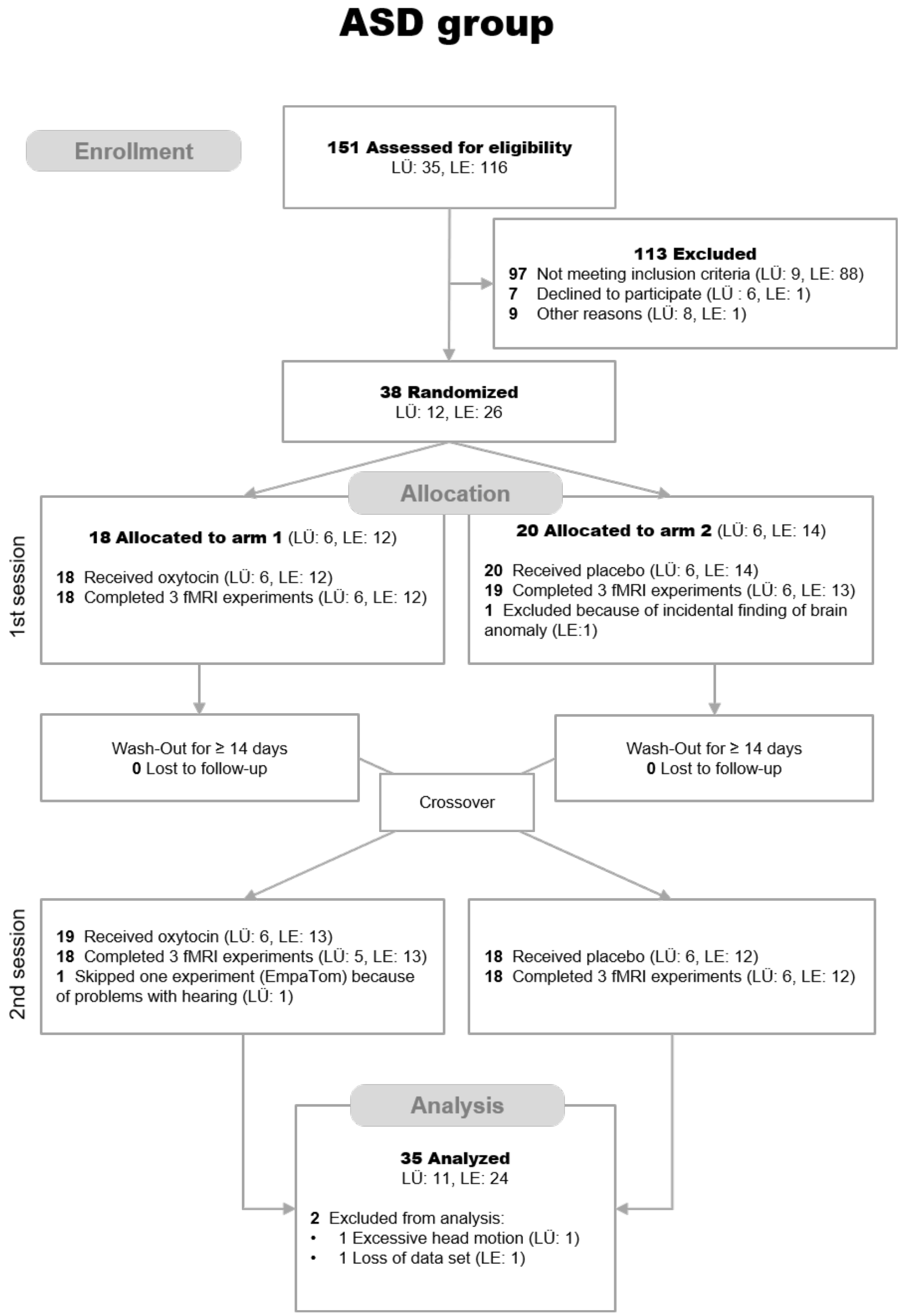

Figure S1: CONSORT flow diagram for the ASD group. LÜ = Lübeck, LE = Leipzig. The number of participants indicated in the “Analysis” section refers to those included in the fMRI analyses.

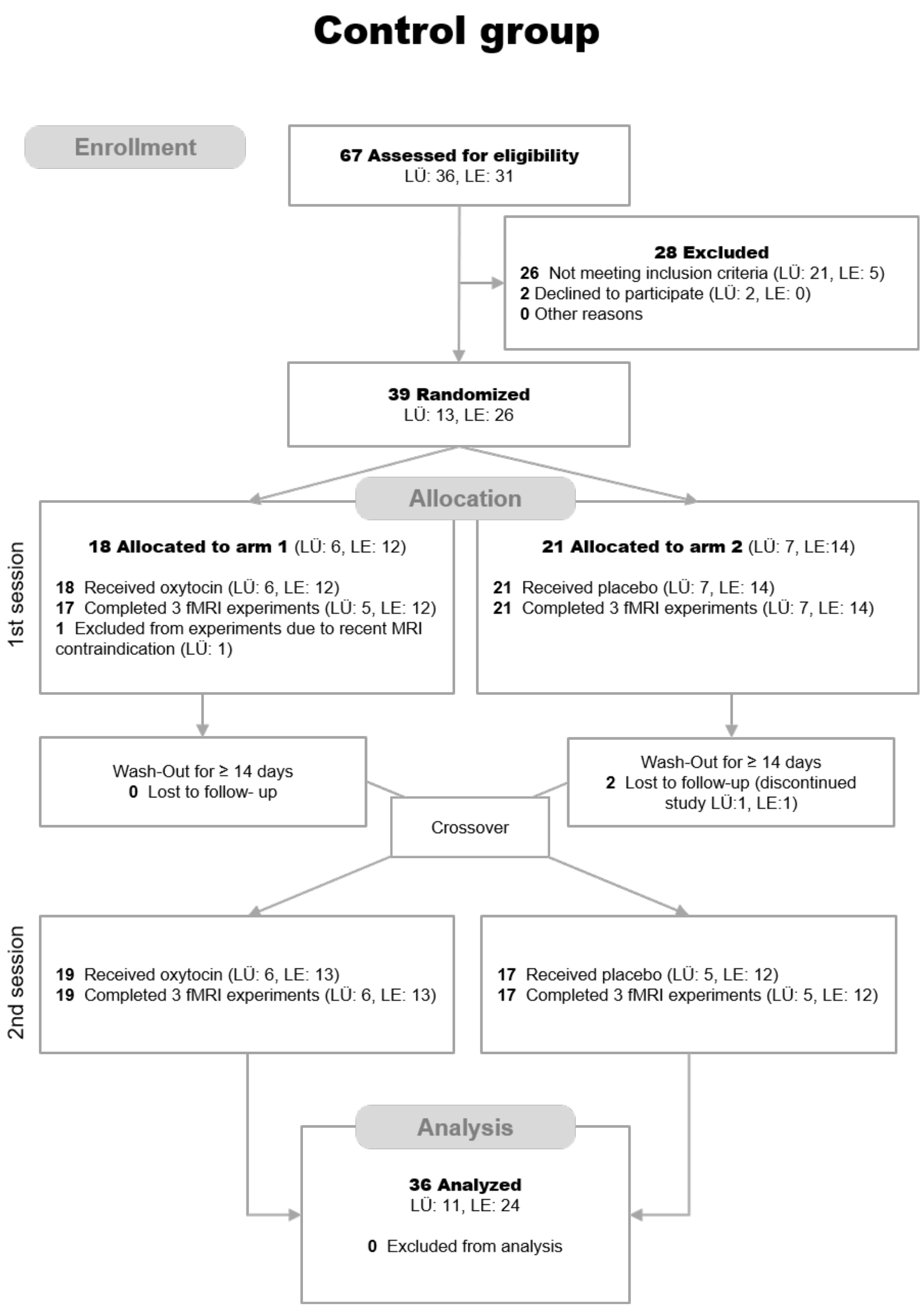

Figure S2: CONSORT flow diagram for the Control group. LÜ = Lübeck, LE = Leipzig. The number of participants indicated in the "Analysis" section refers to those included in the fMRI analyses.

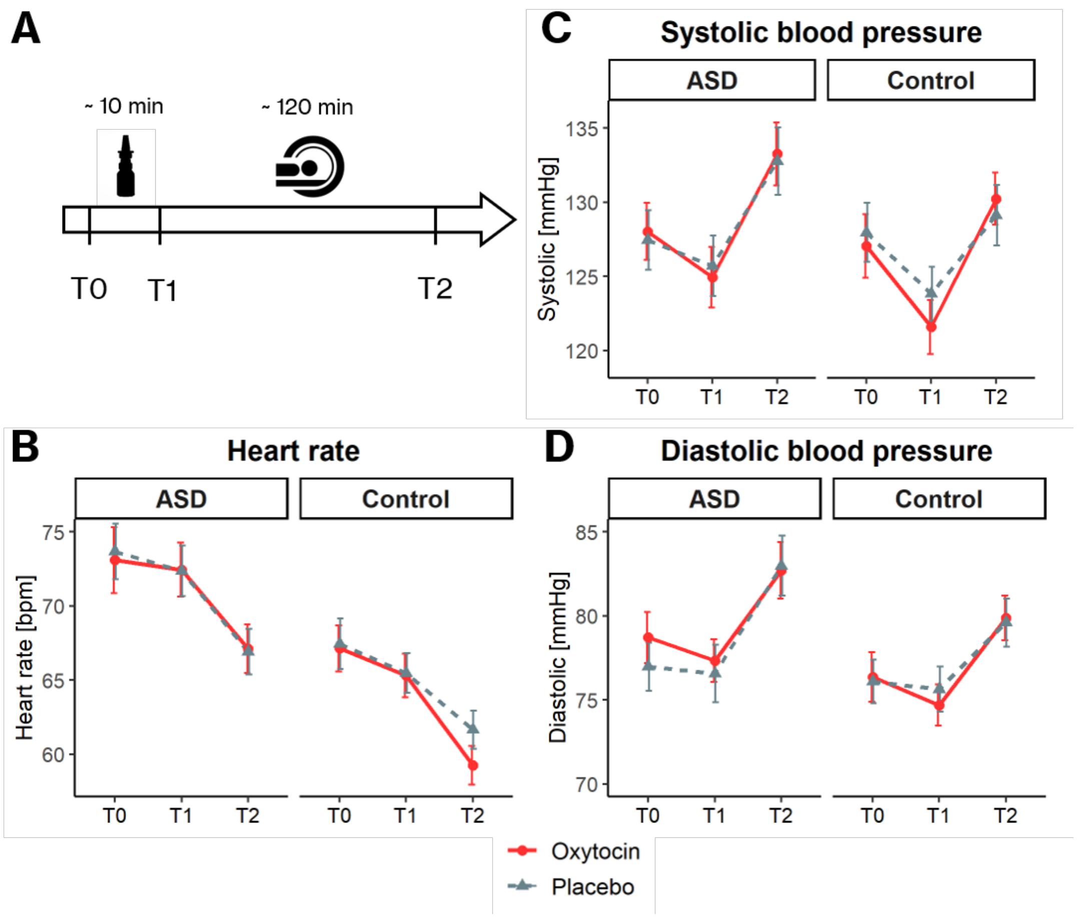

Figure S3: Mean blood pressure and heart rate in ASD and control groups. **A:** Timeline of blood pressure and heart rate measurements. At each timepoint, blood pressure and heart rate were measured twice consecutively and averaged for further analyses, starting with two baseline measurements at the beginning of each visit. The second timepoint was immediately after nasal spray administration, which was about ten minutes after the baseline measurements. The last timepoint was after completion of the MRI measurements (~120 minutes including preparations and a 40-minute waiting period between nasal spray and start of the first experiment). **B:** Mean (±SEM) heart rates. There was a main effect of time (repeated-measures ANOVA with factors group, time, and treatment; main effect time: $F(1.39,97.03) = 46.77$, $p < .001$; $\eta_p^2 = .401$), indicating a significant decrease of heart rate between each time point (post-hoc t-tests: all comparisons $p < .001$). A main effect of group indicated that the ASD group had, on average, higher heart rates than the control group ($F(1, 70) = 11.07$; $p = .001$, $\eta_p^2 = .137$). There were no significant treatment effects. **C:** Mean (±SEM) systolic blood pressure. There was a main effect of time ($F(1.55,108.69) = 45.06$, $p < .001$; $\eta_p^2 = .392$), and systolic blood pressure was significantly different at each time point (post-hoc t-tests: all comparisons $p < .001$). There were no significant group or treatment effects. **D:** Mean (±SEM) diastolic blood pressure. There was a main effect of time ($F(1.62,113.05) = 45.06$, $p < .001$; $\eta_p^2 = .377$), and diastolic blood pressure was significantly different at each time point (post-hoc t-tests: all comparisons $p < .05$). There were no significant group or treatment effects.

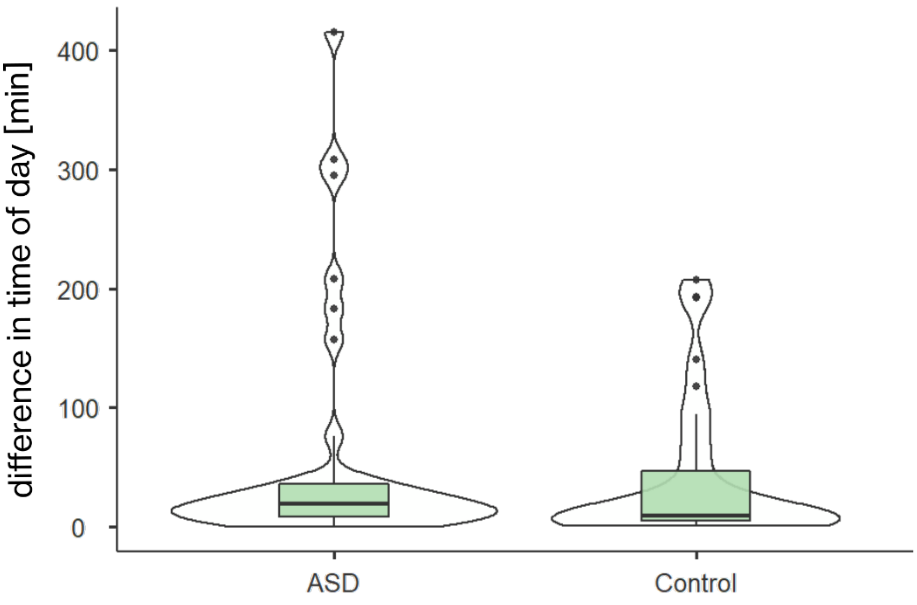

Figure S4: Differences in times of day of nasal spray administration during the two MRI sessions. To avoid systematic time-of-day effects, we aimed to conduct the first and second MRI sessions at the same time of day for each individual participant. This was achieved in 86.3% of cases. In 69.9% of the conducted sessions, participants received the nasal spray between 8:00 am and 12:00 pm, in 24.4% of cases they received it between 12:00 pm and 4:00 pm, and in 5.5% after 4:00 pm.

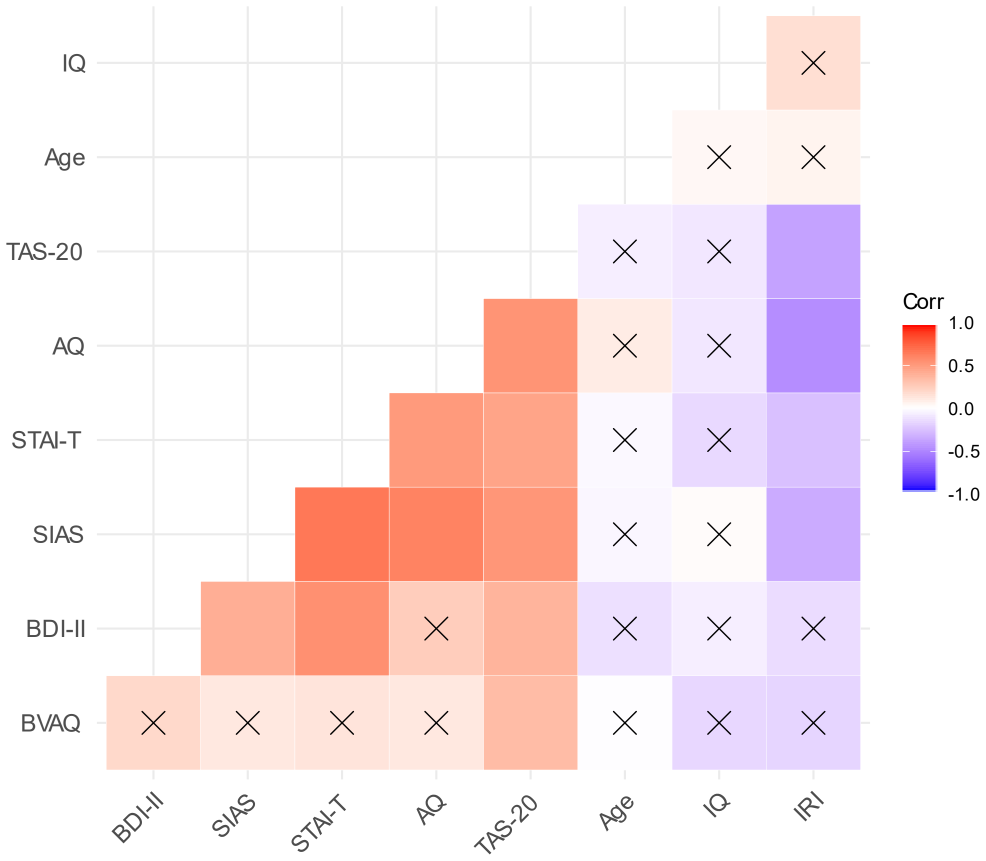

Figure S5: Intercorrelations of age, IQ, mental health, and personality scores. Global IQ was measured using the Wechsler Adult Intelligence Scale (WAIS-IV) (5). AQ = Autism Spectrum Quotient (3), TAS-20 = Toronto Alexithymia Scale (14, 15), BVAQ = Bermond-Vorst Alexithymia Questionnaire (29), BDI-II = Beck Depression Inventory (16, 17), IRI = empathy score (sum score excluding "personal distress" subscale) of the Interpersonal Reactivity Index (25, 26), SIAS = Social Interaction Anxiety Scale (27), STAI-T = State-Trait Anxiety Inventory – trait (9). Spearman correlation coefficients based on data from *N* = 37 ASD participants and *N* = 36 control participants. Non-significant correlations are marked with an x.

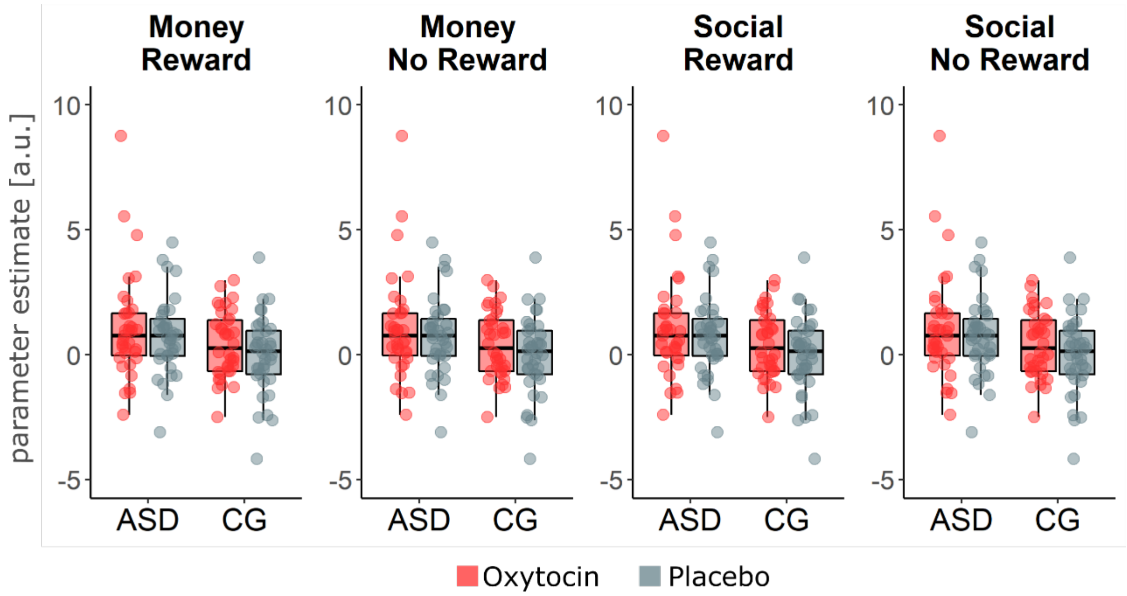

Figure S6: Parameter estimates for all cues against baseline in bilateral ventral striatum. CG = control group, a.u. = arbitrary units. On average, all cues induced significant activation of the ventral striatum compared to baseline (Money – Reward: $t(70) = 3.14$, $p = .001$, $d = 0.41$; Money – No Reward: $t(70) = 3.87$, $p < .001$, $d = 0.46$; Social – Reward: $t(70) = 3.46$, $p < .001$, $d = 0.41$; Social – No Reward: $t(70) = 3.59$, $p < .001$, $d = 0.43$) . Participants with ASD exhibited stronger responses to all cues compared to control participants. There were no significant treatment effects or treatment × group interaction effects (for details, see Supplementary Analyses: Exploratory analyses of cue-related activation in ventral striatum).

SUPPLEMENTARY TABLES

Table S1: Mean latencies between nasal spray administration and start of reward experiment.

	ASD		Control	
Order	*N*	Latency [min] (*M* ± *SD*)	*N*	Latency [min] (*M* ± *SD*)
1	12	40.4 ± 6.47	12	37.8 ± 5.22
2	13	62.7 ± 8.93	13	60.3 ± 10.7
3	12	88.1 ± 4.43	11	87.9 ± 10.3

Note. The monetary and social incentive delay paradigm (MID/SID) was one of three experiments that participants performed in the MRI. The order of the three experiments was randomized. This led to variance in latencies between nasal spray administration and start of the incentive delay paradigm. There were no statistically significant differences between mean latencies of ASD patients and control participants (two-way ANOVA with factors order and group; main effect group: p = .364).

Table S2: Hit rates for each task condition.

	ASD			Control		
	M	Median	*SD*	*M*	Median	*SD*
money no reward	64.6%	63.9%	5.1%	63.3%	63.9%	6.0%
money reward	66.0%	63.9%	3.8%	64.8%	66.7%	5.4%
social no reward	64.6%	66.7%	6.3%	64.2%	63.9%	5.7%
social reward	65.8%	66.7%	5.4%	65.2%	65.3%	4.5%

Note. Task difficulty was adapted to each participant's mean response time to achieve a hit rate of ~66% for each task condition. A first estimate of individual response times was calculated during a training phase at the beginning of the experiment, and the time window for responses was continuously adjusted to the participant's performance during the main experiment. N = 73.

Table S3: Results of repeated measures GLMs on mean response times.

		NHST			Bayes
		F	*p*	η^2_p	BF_{incl}
Main effects	Intensity	20.612	< .001	0.235	79.068
	Task	3.501	0.066	0.05	0.662
	Treatment	0.266	0.608	0.004	0.133
	Group	< .001	0.985	< .001	0.368
Interactions	Treatment × Task	0.002	0.968	< .001	0.168
	Treatment × Intensity	1.243	0.269	0.018	0.189
	Treatment × Group	0.84	0.363	0.012	0.631
	Task × Intensity	0.503	0.481	0.007	0.181
	Task × Group	0.986	0.324	0.014	0.173
	Intensity × Group	0.783	0.379	0.012	0.158
	Treatment ×Task × Intensity	2.321	0.132	0.033	0.249
	Group ×Task × Intensity	3.338	0.072	0.047	0.357

Note. NHST = null hypothesis significance testing. All models include site, arm, and order of the reward experiment within the three sub-experiments. BF_{incl} indicates the Inclusion Bayes factor across matched models as implemented in JASP 0.14.1 (40). This procedure compares models that contain the effect to equivalent models stripped of the effect. Higher-order interactions are excluded. A BF_{incl} >1 indicates relative evidence for the effect of interest, a BF <1 indicates relative evidence against the effect.

Table S4: Correlations between response times and average ventral striatum activation in the anticipation phase.

	Left ventral striatum		Right ventral striatum	
	ρ	*p*	ρ	*p*
Money: No reward	-.226	.030	-.244	.021
Money: Reward	-.125	.152	-.114	.174
Social: No reward	-.197	.052	-.224	.031
Social: Reward	-.205	.044	-.223	.032

Note. Results of a semipartial correlation analysis correcting for site effects in mean response times. We used Spearman's rho (ρ) as a non-parametric measure of rank correlation. We report *p*-values of one-sided tests, assuming negative correlations between response times and ventral striatum activation. *N* = 71.

Table S5: Average task related whole-brain activation in the anticipation phase.

Anatomical region	Cyto area	Side	Cluster size	MNI coordinates			*F*	*p*(FWE)
				x	y	z		
Reward > No reward								
Inferior occipital gyrus	hOc1 [V1]	R	130	24	-97	-1	74.1	< .001
Middle occipital gyrus	hOc3d [V3d]	L	212	-24	-97	-1	70.0	< .001
Fusiform gyrus	FG3	R	2	30	-55	-10	26.8	.019
Fusiform gyrus	hOc4v [V4(v)]	L	3	-30	-79	-13	25.4	.033
Fusiform gyrus	FG3	L	1	-27	-58	-13	25.1	.035
Social > money								
Superior occipital gyrus	hOc2 [V2]	L	142	-9	-97	11	87.4	< .001
Cuneus	hOc2 [V2]	R	103	15	-94	11	75.3	< .001
Lingual gyrus	hOc1 [V1]	L	57	0	-79	-7	58.2	< .001
Inferior occipital gyrus	hOc4lp	R	60	33	-85	-1	46.8	< .001
Fusiform gyrus	FG3	L	11	-30	-58	-10	41.4	< .001
Inferior occipital gyrus	hOc4lp	L	29	-30	-88	-7	38.4	< .001
Fusiform gyrus	FG3	R	3	30	-61	-10	27.9	.014

Note. Results of contrasts of interest in the anticipation phase, averaged across both groups (ASD and control) and treatments (oxytocin and placebo). P-values are family-wise error (FWE) corrected for whole-brain analyses at the voxel level. The “Cyto Area”-column indicates the cytoarchitectonical area as assigned by the SPM Anatomy toolbox v2.2b (59) if available. Anatomical labels were derived respectively. L = left, R = right. *N* = 71.

Table S6: Average task related whole-brain activation in the outcome phase.

Anatomical region	Cyto area	Side	Cluster size	MNI coordinates			*F*	*p*(FWE)
				x	y	z		
Reward > no reward								
Middle occipital gyrus	hOc4lp	R	616	30	-94	2	106.5	< .001
Fusiform gyrus	FG1	R		33	-64	-10	36.7	< .001
Middle occipital gyrus	hOc4lp	L	749	-27	-91	-4	91.9	< .001
Fusiform gyrus	FG1	L		-33	-64	-13	41.8	< .001
Middle occipital gyrus		R	65	30	-64	32	33.4	.002
Angular gyrus		R		27	-61	47	30.2	.005
Superior parietal lobule	7A (SPL)	L	34	-24	-64	53	31.4	.003
Social > money								< .001
Lingual gyrus		R	3580	27	-49	-10	345.0	< .001
Fusiform gyrus	FG3	L		-27	-52	-13	295.4	< .001
Middle occipital gyrus		R		36	-82	14	208.4	< .001
Middle occipital gyrus	hOc2 [V2]	L	316	-12	-103	5	127.4	< .001
Cuneus	hOc2 [V2]	R	213	15	-100	11	88.7	< .001
Mid orbital gyrus	Fp2	R	265	3	53	-13	86.5	< .001
Amygdala	Amygdala (SF)	R	120	21	-4	-16	72.0	< .001
Temporal Pole		R		30	11	-28	26.0	.024
Hippocampus	Amygdala (SF)	L	75	-18	-7	-16	71.6	< .001
Amygdala		L		-30	-4	-19	26.2	.022
Precuneus		R	213	3	-52	26	55.7	< .001
WM		L		-15	-46	35	25.9	.025
Fusiform gyrus	FG4	R	62	42	-46	-22	55.6	< .001
Superior temporal gyrus		R	168	48	-37	8	44.2	< .001
Middle temporal gyrus	PGp (IPL)	R		57	-61	17	33.3	.002
Superior medial gyrus		R	151	6	56	23	42.2	< .001
Superior medial gyrus		L		-6	56	29	39.3	< .001
	hOc1 [V1]	R	19	3	-79	-10	36.9	< .001
Superior parietal lobule	7PC (SPL)	R	32	33	-46	59	31.8	.003
Temporal Pole		R	26	39	20	-31	31.6	.003
IFG (p. orbitalis)		R	9	33	32	-16	31.4	.003
Inferior occipital gyrus	hOc4la	R	3	51	-76	-1	29.4	.007
Postcentral gyrus	Area 2	L	9	-39	-34	41	28.2	.011
Middle temporal gyrus		L	5	-60	-4	-19	26.0	.024

Table S6 (continued).

Anatomical region	Cyto area	Side	Cluster size	MNI coordinates			F	p(FWE)
				x	y	z		
Task x intensity								
Lingual gyrus	hOc3v	R	296	24	-91	-7	85.9	< .001
Fusiform gyrus	FG1	R		33	-64	-10	32.0	.003
Inferior occipital gyrus	hOc4v [V4(v)]	L	270	-27	-88	-10	84.0	< .001
Angular gyrus	hIP3 (IPS)	R	45	30	-58	47	34.6	.001
Cuneus	hOc3d [V3d]	R	19	9	-85	23	29.3	.007
Fusiform gyrus	FG3	L	9	-33	-58	-10	27.3	.015

Note. Results of contrasts of interest in the outcome phase, averaged across both groups (ASD and control) and treatments (oxytocin and placebo). P-values are family-wise error (FWE) corrected for whole-brain analyses at the voxel level. The “Cyto Area”-column indicates the cytoarchitectonical area as assigned by the SPM Anatomy toolbox v2.2b (59) if available. Anatomical labels were derived respectively. L = left, R = right. *N* = 71.

Table S7: Mean stimulus ratings.

	No Reward		Reward			
	M	*SD*	*M*	*SD*	*p*	*d*
Money	2.01	1.25	4.91	2.39	< .001	-1.315
Social	3.12	1.5	5.34	2.14	< .001	-1.193

Note. Participants rated the subjective reward value of all stimuli used in the reward task in an online survey after completion of the last study visit. Answers ranged from 1 (not at all rewarding) to 9 (very strongly rewarding). Due to the large number of missing datasets (63%), we did not include the ratings in our analyses of behavioral and brain imaging data. *N* = 27.

Table S8: Correlations of behavioral reward sensitivity and individual difference variables across all participants after oxytocin and placebo.

	Money			Social		
	Oxytocin	Placebo	*p*(comparison)	Oxytocin	Placebo	*p*(comparison)
Age	.070	.136	.654	-.193	.074	.099
IQ	-.045	.027	.629	.041	-.049	.579
AQ	-.224	.006	.118	.014	-.064	.630
TAS-20	.039	.001	.800	.062	-.038	.537
BVAQ	.060	**.244**	.209	.096	.143	.768
BDI-II	.187	**.232**	.757	.086	-.044	.423
IRI	.086	-.047	.369	.205	.121	.599
SIAS	-.163	.081	.099	.029	-.159	.245
STAI-T	.040	.087	.754	.136	-.125	.108

Note. Reward sensitivity was defined as the difference in mean response times between “no reward” and “reward” trials. Positive values for reward sensitivity imply faster responses to “reward” than “no reward” cues. Correlation coefficients shown here are Spearman rhos, significant correlations ($p < .05$ uncorrected) are highlighted in bold. P-values for comparisons of correlations are reported at an uncorrected level and were calculated using Steiger’s approach for dependent overlapping correlations (50) as implemented in the cocor R package (51). Global IQ was measured using the Wechsler Adult Intelligence Scale (WAIS-IV) (5). AQ = Autism Spectrum Quotient (3), TAS-20 = Toronto Alexithymia Scale (14, 15), BVAQ = Bermond-Vorst Alexithymia Questionnaire (29), BDI-II = Beck Depression Inventory (16, 17), IRI = empathy score (sum score excluding “personal distress” subscale) of the Interpersonal Reactivity Index (25, 26), SIAS = Social Interaction Anxiety Scale (27), STAI-T = State-Trait Anxiety Inventory – trait (9). $N = 73$.

Table S9: Correlations of reward sensitivity in the ventral striatum and individual difference variables across all participants in the anticipation phase.

	Left ventral striatum			Right ventral striatum		
	Oxytocin	Placebo	*p*(comparison)	Oxytocin	Placebo	*p*(comparison)
Money (reward > no reward)						
Age	.008	.188	.302	.151	.199	.788
IQ	.035	-.122	.372	.183	-.149	.064
AQ	**.249**	-.082	.056	**.336**	-.037	.034
TAS-20	.198	-.172	.034	.125	-.097	.221
BVAQ	-.134	-.020	.513	-.157	.016	.338
BDI-II	.157	-.205	.037	.142	-.203	.055
IRI	-.124	.143	.127	-.031	.031	.732
SIAS	**.383**	-.053	.010	**.310**	-.045	.045
STAI-T	**.244**	.050	.260	**.275**	.045	.193
Social (reward > no reward)						
Age	.053	.055	.988	.044	.039	.979
IQ	.079	.136	.729	.176	.107	.687
AQ	.041	.087	.783	.000	.052	.769
TAS-20	.086	.145	.721	.016	.210	.263
BVAQ	-.001	-.154	.357	-.047	-.179	.447
BDI-II	-.092	.111	.224	-.138	.161	.087
IRI	-.114	.010	.458	-.043	-.011	.856
SIAS	-.015	.089	.533	-.033	.093	.471
STAI-T	.006	.062	.738	-.073	.032	.550

Note. Reward sensitivity was defined as ventral striatum activation associated with “reward” cues compared to “no reward” cues. Correlation coefficients shown here are Spearman rhos, significant correlations ($p < .05$ uncorrected) are highlighted in bold. P-values for comparisons of correlations are reported at an uncorrected level and were calculated using Steiger’s approach for dependent overlapping correlations (50) as implemented in the cocor R package (51). Global IQ was measured using the Wechsler Adult Intelligence Scale (WAIS-IV) (5). AQ = Autism Spectrum Quotient (3), TAS-20 = Toronto Alexithymia Scale (14, 15), BVAQ = Bermond-Vorst Alexithymia Questionnaire (29), BDI-II = Beck Depression Inventory (16, 17), IRI = empathy score (sum score excluding “personal distress” subscale) of the Interpersonal Reactivity Index (25, 26), SIAS = Social Interaction Anxiety Scale (27), STAI-T = State-Trait Anxiety Inventory – trait (9). $N = 71$.

Table S10: Correlations of reward sensitivity of the amygdala and individual difference variables across all participants in the outcome phase.

	Left amygdala			Right amygdala		
	Oxytocin	Placebo	*p*(comparison)	Oxytocin	Placebo	*p*(comparison)
Money (reward > no reward)						
Age	-.037	.078	.530	-.111	.019	.469
IQ	-.144	.000	.433	-.111	-.082	.873
AQ	-.105	-.118	.940	.029	-.006	.847
TAS-20	.143	-.146	.115	.228	-.009	.185
BVAQ	.140	.131	.964	.105	.084	.910
BDI-II	.146	-.062	.259	.119	-.021	.438
IRI	.159	.039	.516	.125	-.003	.480
SIAS	.058	-.073	.477	.114	-.034	.414
STAI-T	-.028	-.128	.585	.039	-.067	.560
Social (reward > no reward)						
Age	.126	.003	.453	.024	-.034	.751
IQ	-.030	.221	.123	.006	.107	.575
AQ	.100	.026	.652	.169	.004	.359
TAS-20	.057	-.005	.704	.153	.012	.435
BVAQ	-.092	-.004	.595	-.234	.022	.151
BDI-II	.086	-.103	.249	.010	-.112	.500
IRI	-.142	-.220	.624	-.078	-.213	.448
SIAS	.127	.081	.779	.165	.174	.955
STAI-T	.187	-.055	.138	.105	.104	.993

Note. Reward sensitivity was defined as amygdala activation associated with "reward" outcomes compared to "no reward" outcomes. Correlation coefficients shown here are Spearman rhos. No correlation was statistically significant ($p < .05$ uncorrected). P-values for comparisons of correlations are reported at an uncorrected level and were calculated using Steiger's approach for dependent overlapping correlations (50) as implemented in the cocor R package (51). Global IQ was measured using the Wechsler Adult Intelligence Scale (WAIS-IV) (5). AQ = Autism Spectrum Quotient (3), TAS-20 = Toronto Alexithymia Scale (14, 15), BVAQ = Bermond-Vorst Alexithymia Questionnaire (29), BDI-II = Beck Depression Inventory (16, 17), IRI = empathy score (sum score excluding "personal distress" subscale) of the Interpersonal Reactivity Index (25, 26), SIAS = Social Interaction Anxiety Scale (27), STAI-T = State-Trait Anxiety Inventory – trait (9). $N = 71$.

Table S11: Sensitivity analysis for Bayesian analyses of ventral striatum activation in the anticipation phase.

		Left ventral striatum						Right ventral striatum					
Contrast of interest	Model/Effect	Narrow prior		Default prior		Wide prior		Narrow prior		Default prior		Wide prior	
		BF_{10}	BF_{incl}	BF_{10}	BF_{incl}	BF_{10}	BF_{incl}	BF_{10}	BF_{incl}	BF_{10}	BF_{incl}	BF_{10}	BF_{incl}
Reward > No Reward	Group	0.732	0.732	0.453	0.453	0.252	0.252	0.983	0.983	0.661	0.661	0.376	0.376
	Treatment	0.452	0.452	0.226	0.226	0.122	0.122	0.373	0.373	0.191	0.191	0.092	0.092
	Group × Treatment	0.167	0.501	0.030	0.295	0.004	0.131	0.301	0.782	0.063	0.530	0.011	0.309
Social > Money	Group	0.429	0.429	0.208	0.208	0.114	0.114	0.437	0.437	0.218	0.218	0.115	0.115
	Treatment	0.480	0.480	0.256	0.256	0.132	0.132	0.512	0.512	0.260	0.260	0.139	0.139
	Group × Treatment	0.103	0.511	0.013	0.256	0.002	0.137	0.121	0.536	0.018	0.046	0.003	0.013
Task × Intensity	Group	0.482	0.482	0.236	0.236	0.120	0.120	0.432	0.432	0.212	0.212	0.114	0.114
	Treatment	0.463	0.463	0.249	0.249	0.131	0.131	0.469	0.469	0.239	0.239	0.138	0.138
	Group × Treatment	0.370	1.599	0.082	1.448	0.014	0.867	0.303	1.466	0.057	1.131	0.010	0.706

Note. Bayesian analyses reported in the main text (using the default prior: r = 0.5 for fixed effects) were re-run using a narrower (r = 0.2 for fixed effects) and wider prior (r = 1 for fixed effects) to assess the sensitivity of our analyses to prior specifications (60). Since our models only contain fixed effects and no random effects or covariates, only the hyperparameter for fixed effects was changed. BF_{incl} indicates the Inclusion Bayes factor across matched models as implemented in JASP 0.14.1 (40).

Table S12: Sensitivity analysis for Bayesian analyses of amygdala activation in the outcome phase.

		Left amygdala						Right amygdala					
Contrast of interest	Model/Effect	Narrow prior		Default prior		Wide prior		Narrow prior		Default prior		Wide prior	
		BF_{10}	BF_{incl}	BF_{10}	BF_{incl}	BF_{10}	BF_{incl}	BF_{10}	BF_{incl}	BF_{10}	BF_{incl}	BF_{10}	BF_{incl}
Reward > No Reward	Group	0.441	0.441	0.218	0.218	0.144	0.144	0.518	0.518	0.275	0.275	0.146	0.146
	Treatment	0.519	0.519	0.267	0.267	0.120	0.120	0.411	0.411	0.193	0.193	0.105	0.105
	Group * Treatment	0.118	0.489	0.016	0.287	0.002	0.013	0.108	0.51	0.014	0.271	0.003	0.18
Social > Money	Group	0.663	0.663	0.374	0.374	0.210	0.210	0.625	0.625	0.456	0.456	0.203	0.203
	Treatment	0.694	0.694	0.387	0.387	0.211	0.211	0.412	0.412	0.204	0.204	0.107	0.107
	Group * Treatment	0.217	0.146	0.038	0.266	0.006	0.142	0.150	0.582	0.022	0.288	0.004	0.172
Task * Intensity	Group	0.459	0.459	0.226	0.226	0.126	0.126	0.434	0.434	0.221	0.221	0.122	0.122
	Treatment	0.694	0.694	0.380	0.380	0.211	0.211	0.682	0.682	0.384	0.384	0.207	0.207
	Group * Treatment	0.533	1.663	0.116	1.297	0.024	0.909	0.950	3.292	0.245	3.017	0.05	2.141

Note. Bayesian analyses reported in the main text (using the default prior: r = 0.5 for fixed effects) were re-run using a narrower (r = 0.2 for fixed effects) and wider prior (r = 1 for fixed effects) to assess the sensitivity of our analyses to prior specifications (60). Since our models only contain fixed effects and no random effects or covariates, only the hyperparameter for fixed effects was changed. BF_{incl} indicates the Inclusion Bayes factor across matched models as implemented in JASP 0.14.1 (40).

SUPPLEMENTARY REFERENCES

1. Lord C, Risi S, Lambrecht L, Cook EH, Leventhal BL, DiLavore PC, *et al.* (2000): Autism Diagnostic Observation Schedule (ADOS). *J Autism Dev Disord*. . doi: 10.1007/BF02211841.

2. Rutter M, Le Couteur A, Lord C (2003): *Autism Diagnostic Interview-Revised (ADI-R)*. Los Angeles: Western Psychological Services.

3. Baron-Cohen S, Wheelwright S, Skinner R, Martin J, Clubley E (2001): The Autism-Spectrum Quotient (AQ): Evidence from Asperger syndrome/high-functioning autism, males and females, scientists and mathematicians. *J Autism Dev Disord*. 31: 5–17.

4. Preckel K, Kanske P, Singer T, Paulus FM, Krach S (2016): Clinical trial of modulatory effects of oxytocin treatment on higher-order social cognition in autism spectrum disorder: a randomized, placebo-controlled, double-blind and crossover trial. *BMC Psychiatry*. 16: 329.

5. Wechsler D (2008): *Wechsler Adult Intelligence Scale—Fourth Edition*. San Antonio, TX: Pearson Assessment.

6. Kanske P, Böckler A, Trautwein FM, Singer T (2015): Dissecting the social brain: Introducing the EmpaToM to reveal distinct neural networks and brain-behavior relations for empathy and Theory of Mind. *Neuroimage*. 122: 6–19.

7. Hariri AR, Tessitore A, Mattay VS, Fera F, Weinberger DR (2002): The amygdala response to emotional stimuli: A comparison of faces and scenes. *Neuroimage*. 17: 317–323.

8. Preckel K, Trautwein FM, Paulus FM, Kirsch P, Krach S, Singer T, Kanske P (2019): Neural mechanisms of affective matching across faces and scenes. *Sci Rep*. 9. doi: 10.1038/s41598-018-37163-9.

9. Laux L, Glanzmann P, Schaffner P, Spielberger CD (1981): *Das State-Trait-Angstinventar (STAI) : theoretische Grundlagen und Handanweisung*. Weinheim: Beltz.

10. Kamp-Becker I, Poustka L, Bachmann C, Ehrlich S, Hoffmann F, Kanske P, *et al.* (2017): Study protocol of the ASD-Net, the German research consortium for the study of Autism Spectrum Disorder across the lifespan: From a better etiological understanding, through valid diagnosis, to more effective health care. *BMC Psychiatry*. 17: 1–14.

11. Bauer M, Banaschewski T, Heinz A, Kamp-Becker I, Meyer-Lindenberg A, Padberg F, *et al.* (2016): The German research network for mental disorders. *Nervenarzt*. 87: 989–1010.

12. Oldfield RC (1971): The assessment and analysis of handedness: The Edinburgh Inventory. *Neuropsychologia*. 9: 97–113.

13. Derogatis LR (1983): The Brief Symptom Inventory: An Introductory Report. *Psychol Med*. 13: 595–605.

14. Bach M, Bach D, de Zwaan M, Serim M, Böhmer F (1996): Validation of the German version of the 20-item Toronto Alexithymia Scale in normal persons and psychiatric patients. *Psychother Psychosom Med Psychol*. 46: 23–8.

15. Bagby RM, Parker JDA, Taylor GJ (1994): The twenty-item Toronto Alexithymia scale—I. Item selection and cross-validation of the factor structure. *J Psychosom Res*. 38: 23–32.

16. Beck AT, Steer RA, Brown GK (1996): *Beck Depression Inventory (BDI-II)*, 2nd ed. San Antonio, TX: The Psychological Corporation.

17. Kühner C, Bürger C, Keller F, Hautzinger M (2007): Reliabilität und Validität des revidierten Beck-Depressions-inventars (BDI-II). Befunde aus deutschsprachigen Stichproben. *Nervenarzt*. 78: 651–656.

18. Tombaugh TN (2004): Trail Making Test A and B: Normative data stratified by age and education. *Arch Clin Neuropsychol*. 19: 203–214.

19. Schmidt K-H, Metzler P (1992): *Wortschatztest - WST*. Göttingen: Hogrefe.

20. World Health Organization (2001): *WHO Disability Assessment Schedule 2.0 (WHODAS 2.0)*. . Retrieved from https://www.who.int/classifications/icf/whodasii/.

21. Grabe H, Schulz A, Schmidt C, Appel K, Driessen M, Wingenfeld K, *et al.* (2012): A brief instrument for the assessment of childhood abuse and neglect: the childhood trauma screener (CTS). *Psychiatr Prax*. 39: 109–115.

22. Carver CS, White TL (1994): Behavioral inhibition, behavioral activation, and affective responses to impending reward

and punishment: the BIS/BAS scales. *J Pers Soc Psychol*. 67: 319.

23. Spinella M (2007): Normative data and a short form of the Barratt Impulsiveness Scale. *Int J Neurosci*. 117: 359–368.

24. Watson D, Clark LA, Tellegen A (1988): Development and validation of brief measures of positive and negative affect: The PANAS scales. *J Pers Soc Psychol*. 54: 1063–1070.

25. Davis MH (1983): A multidimensional approach to individual differences in empathy. *J Pers Soc Psychol*. 44: 113–126.

26. Paulus C (2009): *Der Saarbrücker Persönlichkeitsfragebogen (SPF-IRI) zur Messung von Empathie*. . Retrieved from http://hdl.handle.net/20.500.11780/3343.

27. Mattick RP, Clarke JC (1998): Development and validation of measures of social phobia scrutiny fear and social interaction anxiety. *Behav Res Ther*. 36: 455–470.

28. Garnefski N, Kraaij V, Spinhoven P (2001): Negative life events, cognitive emotion regulation and emotional problems. *Pers Individ Dif*. 30: 1311–1327.

29. Vorst HCM, Bermond B (2001): Validity and reliability of the Bermond-Vorst Alexithymia Questionnaire. *Pers Individ Dif*. 30: 413–434.

30. The jamovi project (2019): Jamovi. . Retrieved October 13, 2020, from https://www.jamovi.org/.

31. Whelan R (2008): Effective analysis of reaction time data. *Psychol Rec*. 58: 475–482.

32. The Wellcome Centre for Human Neuroimaging (2014): SPM12 Software - Statistical Parametric Mapping. . Retrieved November 2, 2020, from https://www.fil.ion.ucl.ac.uk/spm/software/spm12/.

33. The MathWorks Inc. (2019): MATLAB. . Retrieved from https://www.mathworks.com/.

34. Diekhof EK, Kaps L, Falkai P, Gruber O (2012): The role of the human ventral striatum and the medial orbitofrontal cortex in the representation of reward magnitude - An activation likelihood estimation meta-analysis of neuroimaging studies of passive reward expectancy and outcome processing. *Neuropsychologia*. 50: 1252–1266.

35. Diedrichsen J, Shadmehr R (2005): Detecting and adjusting for artifacts in fMRI time series data. *Neuroimage*. 27: 624–634.

36. Tzourio-Mazoyer N, Landeau B, Papathanassiou D, Crivello F, Etard O, Delcroix N, *et al.* (2002): Automated anatomical labeling of activations in SPM using a macroscopic anatomical parcellation of the MNI MRI single-subject brain. *Neuroimage*. . doi: 10.1006/nimg.2001.0978.

37. Knutson B, Adams CM, Fong GW, Hommer D (2001): Anticipation of increasing monetary reward selectively recruits nucleus accumbens. *J Neurosci*. 21: RC159.

38. Maldjian JA, Laurienti PJ, Kraft RA, Burdette JH (2003): An automated method for neuroanatomic and cytoarchitectonic atlas-based interrogation of fMRI data sets. *Neuroimage*. 19: 1233–1239.

39. Wilson RP, Colizzi M, Bossong MG, Allen P, Kempton M, Abe N, *et al.* (2018): The Neural Substrate of Reward Anticipation in Health: A Meta-Analysis of fMRI Findings in the Monetary Incentive Delay Task. *Neuropsychol Rev*. 28. doi: 10.1007/s11065-018-9385-5.

40. JASP Team (2020): JASP. . Retrieved from https://jasp-stats.org/.

41. Martins D, Rademacher L, Gabay AS, Taylor R, Richey JA, Smith DV, *et al.* (2021): Mapping social reward and punishment processing in the human brain: A voxel-based meta-analysis of neuroimaging findings using the Social Incentive Delay task. *Neurosci Biobehav Rev*. 122: 1–17.

42. Rademacher L, Krach S, Kohls G, Irmak A, Gründer G, Spreckelmeyer KN (2010): Dissociation of neural networks for anticipation and consumption of monetary and social rewards. *Neuroimage*. 49: 3276–3285.

43. Domes G, Heinrichs M, Gläscher J, Büchel C, Braus DF, Herpertz SC (2007): Oxytocin attenuates amygdala responses to emotional faces regardless of valence. *Biol Psychiatry*. 62: 1187–1190.

44. Domes G, Kumbier E, Heinrichs M, Herpertz SC (2014): Oxytocin promotes facial emotion recognition and amygdala reactivity in adults with asperger syndrome. *Neuropsychopharmacology*. 39: 698–706.

45. Love J, Selker R, Marsman M, Jamil T, Dropmann D, Verhagen J, *et al.* (2019): JASP: Graphical Statistical Software

for Common Statistical Designs. *J Stat Softw*. 88: 1–17.

46. Rouder JN, Morey RD, Speckman PL, Province JM (2012): Default Bayes factors for ANOVA designs. *J Math Psychol*. 56: 356–374.

47. Mathôt S (2017, May): Bayes like a Baws: Interpreting Bayesian Repeated Measures in JASP. . Retrieved July 14, 2021, from https://www.cogsci.nl/blog/interpreting-bayesian-repeated-measures-in-jasp.

48. Spielberger CD, Gorsuch RL, Lushene L, Vagg PR, Jacobs GA (1983): *Manual for the State-Trait Anxiety Inventory*. Palo Alto, CA: Consulting Psychologists Press.

49. R Core Team (2020): R: A language and environment for statistical computing. . Retrieved from https://www.r-project.org/.

50. Steiger JH (1980): Tests for comparing elements of a correlation matrix. *Psychol Bull*. 87.

51. Diedenhofen B, Musch J (2015): cocor: A Comprehensive Solution for the Statistical Comparison of Correlations. (J. Olivier, editor) *PLoS One*. 10: e0121945.

52. Holm S (1979): A simple sequentially rejective multiple test procedure. *Scand J Stat*. 6: 65–70.

53. Alaerts K, Bernaerts S, Vanaudenaerde B, Daniels N, Wenderoth N (2019): Amygdala–Hippocampal Connectivity Is Associated With Endogenous Levels of Oxytocin and Can Be Altered by Exogenously Administered Oxytocin in Adults With Autism. *Biol Psychiatry Cogn Neurosci Neuroimaging*. 4: 655–663.

54. Gordon I, Jack A, Pretzsch CM, Vander Wyk B, Leckman JF, Feldman R, Pelphrey KA (2016): Intranasal Oxytocin Enhances Connectivity in the Neural Circuitry Supporting Social Motivation and Social Perception in Children with Autism. *Sci Rep*. 6: 1–14.

55. Watanabe T, Kuroda M, Kuwabara H, Aoki Y, Iwashiro N, Tatsunobu N, *et al.* (2015): Clinical and neural effects of six-week administration of oxytocin on core symptoms of autism. *Brain*. 138. doi: 10.1093/brain/awv249.

56. Paulus FM, Krach S, Bedenbender J, Pyka M, Sommer J, Krug A, *et al.* (2013): Partial support for ZNF804A genotype-dependent alterations in prefrontal connectivity. *Hum Brain Mapp*. 34: 304–313.

57. Paulus FM, Bedenbender J, Krach S, Pyka M, Krug A, Sommer J, *et al.* (2014): Association of rs1006737 in CACNA1C with alterations in prefrontal activation and fronto-hippocampal connectivity. *Hum Brain Mapp*. 35: 1190–1200.

58. Esslinger C, Walter H, Kirsch P, Erk S, Schnell K, Arnold C, *et al.* (2009): Neural Mechanisms of a Genome-Wide Supported Psychosis Variant. *Science (80-)*. 324: 605–605.

59. Eickhoff SB, Stephan KE, Mohlberg H, Grefkes C, Fink GR, Amunts K, Zilles K (2005): A new SPM toolbox for combining probabilistic cytoarchitectonic maps and functional imaging data. *Neuroimage*. 25: 1325–1335.

60. van Doorn J, van den Bergh D, Böhm U, Dablander F, Derks K, Draws T, *et al.* (2020): The JASP guidelines for conducting and reporting a Bayesian analysis. *Psychon Bull Rev 2020 283*. 28: 813–826.
